# Introduced ant species occupy empty climatic niches in Europe

**DOI:** 10.1038/s41598-021-82982-y

**Published:** 2021-02-08

**Authors:** Xavier Arnan, Elena Angulo, Raphaël Boulay, Roberto Molowny-Horas, Xim Cerdá, Javier Retana

**Affiliations:** 1 Universidade de Pernambuco – Campus Garanhuns, Garanhuns, PE 55294-902 Brazil; 2 CREAF, 08193 Cerdanyola del Vallès, Catalunya Spain; 3 Estación Biológica de Doñana, CSIC, Avda Américo Vespucio, 26, 41092 Sevilla, Spain; 4 Institute of Insect Biology, University François Rabelais of Tours, 37200 Tours, France; 5 Univ Autònoma Barcelona, 08193 Cerdanyola del Vallès, Catalunya Spain

**Keywords:** Biodiversity, Community ecology, Ecosystem ecology, Invasive species, Ecology, Ecology

## Abstract

Exploring shifts in the climatic niches of introduced species can provide significant insight into the mechanisms underlying the invasion process and the associated impacts on biodiversity. We aim to test the phylogenetic signal hypothesis in native and introduced species in Europe by examining climatic niche similarity. We examined data from 134 ant species commonly found in western Europe; 130 were native species, and 4 were introduced species. We characterized their distribution patterns using species records from different databases, determined their phylogenetic relatedness, and tested for a phylogenetic signal in their optimal climatic niches. We then compared the introduced species’ climatic niches in Europe with their climatic niches in their native ranges and with the climatic niches of their closest relative species in Europe. We found a strong phylogenetic signal in the optimal climatic niches of the most common ant species in Europe; however, this signal was weak for the main climatic variables that affect the distributions of introduced versus native species. Also, introduced species occupied different climatic niches in Europe than in their native ranges; furthermore, their European climatic niches did not resemble those of their closest relative species in Europe. We further discovered that there was not much concordance between the climatic niches of introduced species in their native ranges and climatic conditions in Europe. Our findings suggest that phylogenetics do indeed constrain shifts in the climatic niches of native European ant species. However, introduced species would not face such constraints and seemed to occupy relatively empty climatic niches.

## Introduction

Species vary enormously in their climatic niches^1^. A species’ ability to adapt to changing environments may be, to some degree, constrained by its evolutionary history^2,3^. In fact, the degree to which ecological niches are conserved across evolutionary time is the topic of intense debate^4^ for two main reasons. First, over the last decade, this issue has aroused increasing interest^5–10^, partly because it informs our understanding of global biodiversity gradients^11^ and partly because it helps us understand how species might adapt to ongoing climate change^3^. Second, the results thus far remain equivocal—evidence exists for both niche conservatism and niche divergence within clades^8^.

Niche conservatism, which is the tendency of species to retain ancestral ecological characteristics^12^, is expected to occur during species diversification^13^. The existence of phylogenetic signals—ecological similarity between species that is linked to phylogenetic relatedness (the phylogenetic signal hypothesis, *sensu*^5^)—in climatic niches provides suggestive, but not conclusive, evidence for the existence of phylogenetic niche conservatism^5^. However, such signals do raise doubt about the alternative hypothesis that niches evolve quickly^14,15^ and independently of phylogeny^6^. Although there is some evidence of phylogenetic signals in the climatic niches of different groups of organisms (plants^16,17^, salamanders^18^; amphibians in general^7^; and *Drosophila*^3^), other studies have found that climatic niches are not phylogenetically conserved (birds^19^; frogs^20^; lizards^21^; mammals in general^6,22^; monkeys^23^; bats^24^), resulting in mixed support for the idea that climatic niches are determined by evolutionary history (for a review, see^5^).

Over the last few decades, many species have spread beyond their natural ranges, with dramatic consequences for biodiversity and conservation^25–29^). Species introductions can provide natural experiments for testing whether a species’ climatic niche in its introduced range is a consequence of phylogenetic constraints, plasticity, or evolutionary shifts in response to novel pressures^30–33^). Moreover, characterizing the climatic niches of invaders can help us better understand the climatic conditions under which local communities could be most vulnerable to invasions^34^. If evolutionary history constrains climatic niches in both native and introduced species, the latter should perform well under the same climatic conditions as their closest relative species in their introduced ranges. In such a case, and since competition should be strong between phylogenetically close species^35^, there could be dramatic consequences for biodiversity conservation. That said, introduced species and their closest relative species in their introduced ranges could display disparate climatic niches given that the two might have evolved in different biogeographical areas and under different climatic conditions; consequently, introduced species could have nearly the same climatic niches as in their native ranges^17^. Alternatively, given that they likely experience evolutionary niche expansion as their introduced ranges expand^31,36,37^ and they face colonization constraints^33,38^, we might expect introduced species to move into new climatic niches, which could be different from those in their native ranges and from those of their closest relative species in their introduced ranges.

In this study, we characterized the climatic niches of native and introduced ant species commonly found in western Europe and the Mediterranean Basin (hereafter, Europe). The aim was to test the phylogenetic signal hypothesis for the climatic niches of these ants and to determine if there was support for any of the above predictions for the introduced species. Ants are dominant organisms in most terrestrial ecosystems, both in terms of biomass and ecological function^39^. Furthermore, invasive ants are some of the most widespread invasive animal species and cause a great deal of damage^40,41^. There are a few studies that have examined support for the phylogenetic signal hypothesis in ants, and they have reached contradictory conclusions^10,42–44^, the same as for other taxa (as mentioned above). Here, we examined data from 134 European ants; we characterized their distribution patterns using species records from different databases and determined their phylogenetic relatedness. We made the following key predictions: 1) there is a phylogenetic signal in the optimal climatic niches of ants in Europe (i.e., closely related species have similar optimal climatic niches); and 2) if evolutionary history constrains ant species evolution, the climatic niches of introduced species will be similar to those of their closest relative species in Europe. In contrast, if phylogeny does not constrain ant climatic niches, we expected two alternative results. First, because introduced species may have evolved under very different conditions than their closest relative species in Europe, we could expect the two groups to have very different climatic niches, and introduced species could occupy nearly the same climatic niches as in their native ranges. Second, introduced species might have experienced a rapid shift in their climatic niches due to selection pressures imposed by the invasion process and thus might occupy climatic niches that are entirely distinct from those in their native ranges or from those of their closest relative species in Europe. If we find evidence for this latter prediction, it would be important to compare and contrast these new niches to gain insight into the eco-evolutionary mechanisms underlying the invasion process and the associated impacts on biodiversity.

## Methods

### Data collection

Species choice was based on the availability of distribution and relatedness data. We used four websites that host extensive databases^45–48^ built from natural history collections to obtain geographical records for European ant species from six subfamilies (Dolichoderinae, Formicinae, Leptanillinae, Myrmicinae, Ponerinae, and Proceratiinae; see Table S1 in the Electronic Supplementary Material). Our initial dataset contained 137 species, which represented the most common ant species in western Europe (non-parasitic species only^49^). The mean number of records per species was 119 (range: 10–739). Latitudes ranged from 12.38º to 68.17º; longitudes ranged from − 18.13º to 73.79º. To attain a reasonable degree of biogeographical consistency in our dataset, we only considered ant species that occur in and around western Europe and the Mediterranean Basin (Figure S1). Records stemming from outside this zone were excluded and occurrences of the same species separated by a distance of < 500 m were also excluded to avoid spatial autocorrelation. Our study zone thus comprised all of western Europe (including the Baltic countries and the Scandinavian Peninsula) plus northern Africa, the Mediterranean coast of Middle Eastern countries, the Anatolian Peninsula, and western Ukraine and Belarus.

### Determination of ant species origin

Using this initial set of 137 species, we separated out species that are native to Europe from species that were introduced into Europe (Table S1). To accomplish this task, we used a published list of the 241 ant species that have become successfully established outside their native ranges^50^. Twenty-nine of the species in our dataset were found on this list. We determined whether or not these 29 ant species were native to Europe using four main resources: to verify the species’ native and introduced ranges^46,48^ as well as to clarify the species’ origins and sites of natural occurrence and introduction in and around Europe^51,52^ (Table S2). Some of the species are native to the Mediterranean Basin but have been introduced into northern Europe; we excluded such records from our dataset because we were interested in the climatic niche of native species in Europe (Table S2; Figure S2). Based on this information, we discovered that 4 of the 29 species had been introduced into Europe. They are *Cardioncondyla emeryi*, *Lasius neglectus*, *Linepithema humile*, and *Pheidole megacephala*. *Monomorium pharaonis* was excluded because it only occurred in urban areas. *Cardiocondyla mauritanica* and *Hypoponera punctatissima* were also excluded due to the uncertainty surrounding their origins, histories, and range expansion patterns (see Table S2). We therefore ended up with a final dataset composed of 134 species (130 native and 4 introduced).

*Linepithema humile* (the Argentine ant) and *P. megacephala* (the big-headed ant) are 2 of the 5 ant species found on the list of the world’s 100 worst invaders, and, together with *L. neglectus* (the invasive garden ant), they are among the 19 species described as highly invasive by the IUCN invasive species specialist group (ISSG)^40,53^. The ISSG has classified them as invasive species because of their documented impacts on biological diversity and/or human activities. Unlike the three others, *C. emeryi* (the sneaking ant) has not been formally classified as an invasive species. It is a highly inconspicuous ant with a cosmopolitan distribution that seems to have had little impact in its introduced range^54^; however, concerns have been raised about its potential effects^55^. Indeed, more recent studies indicate that this species may become invasive in the future, given that its rate of introduction has climbed over the last few decades and that it displays life history traits associated with successful invaders^50,56^. Consequently, *C. emeryi* shares many similarities with ant species that have been classified as invasive by IUCN. However, because it has not yet officially received that designation, we will refer to all four species as “introduced species.”

Although these four species have worldwide distributions, in Europe, there are far fewer records for them than there are for most native European species (Table S1). This fact serves as a strong indicator that they are not widespread within Europe. Furthermore, the locations at which they have been observed provide a clear sense of the climatic conditions under which these species can survive as they continue the invasion process.

### Ant phylogeny

**Figure 1:**
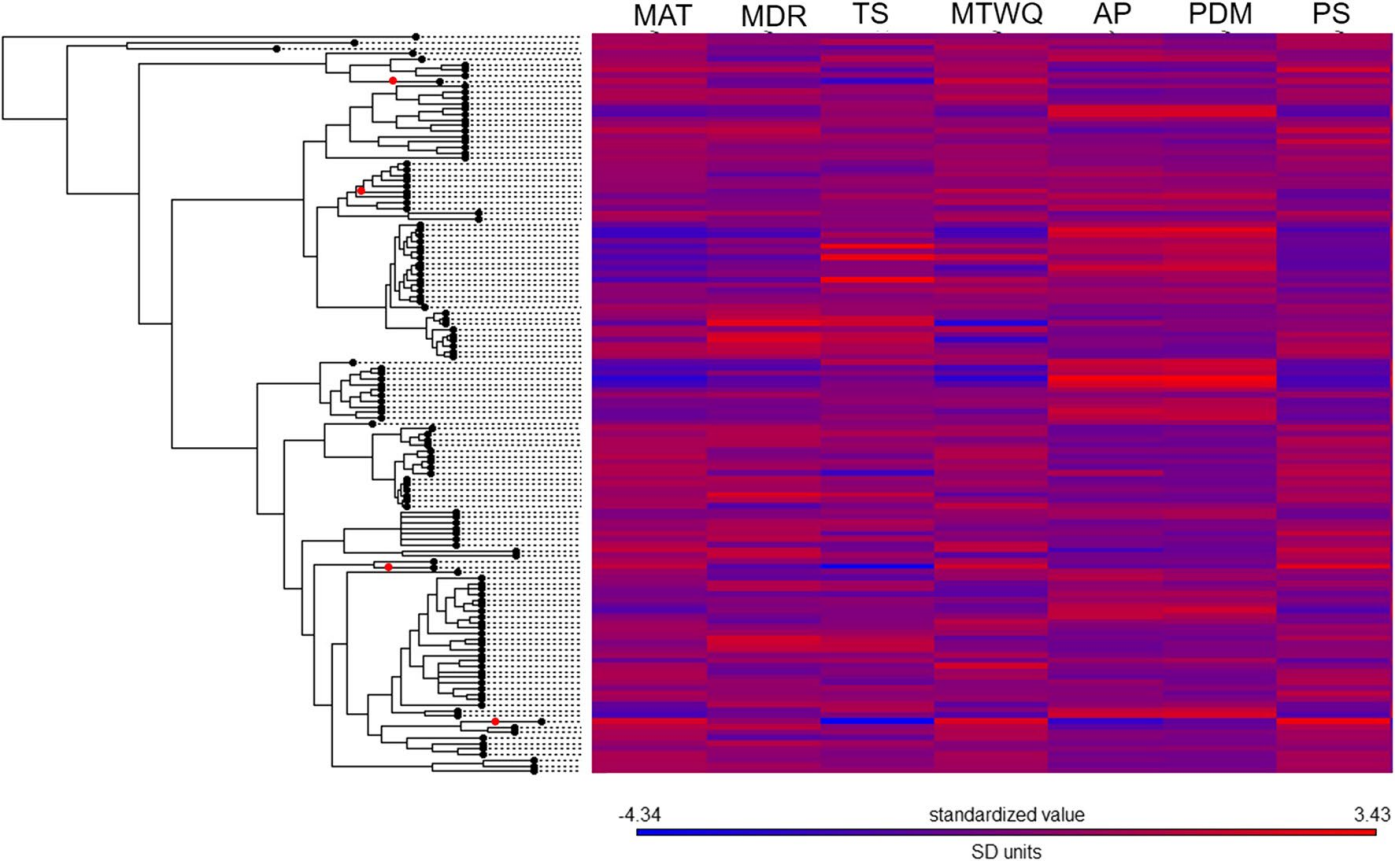
Phylogenetic heat map showing phylogenetic relationships among species and the standardized mean values of the variables associated with the climatic niches. Abbreviations: *MAT* mean annual temperature; MDR mean diurnal range (mean of monthly [max temp − min temp]); *TS* temperature seasonality; *MTWQ* mean temperature of the wettest quarter of the year; *AP* annual precipitation; *PDM* precipitation of driest month; *PS* precipitation seasonality. The introduced species are indicated with red dots.

We created a complete phylogeny that incorporated the 134 ant species (Fig. 1, Figure S3). Right now, there is no complete, species-level ant phylogeny. Thus, we used an approach that incorporated as much information as possible given our understanding of ant relationships. To do so, we began by using a backbone tree derived from a time-calibrated, genus-level phylogeny^57^; however, the phylogenetic relationships within Myrmicinae were taken from Ward et al.^58^. They are the most comprehensive phylogenetic trees to date for ants. This phylogeny was then pruned to keep a single species per genus and thus generate a time-calibrated genus-level phylogeny. We then manually added species to this genus-level tree (by directly editing the NEWICK tree). Within each genus and aiming to determine which species diverged earlier or later, we used information from the literature describing different species-specific phylogenetic relationships based on both molecular and morphological data. While we recognize that, ideally, a phylogeny should be reconstructed solely from molecular data, such data were not universally available. We gathered information from 34 references (Appendix S1); 17 (50%) provided molecular data. We found molecular data for 74 of the 134 species (55%). Since molecular data were not available for the other 60 species, we employed morphological data instead. Then, and because we had only a divergence time or branch length of genera (from Moureau and Bell^57^), we then applied the same divergence time (i.e. the divergence time of that genus divided by the number of nodes within that genus) to all the nodes within a given genus; this divergence time or branch length was determined from the species relationships that we had reconstructed using species-level molecular and morphological data. We believe that this approach is more appropriate than simply treating each species within a genus as a basal species (i.e., each species is as divergent from the others as they all are from the sister genus) or using terminal polytomies (i.e., there is zero divergence time between each species and all the others), which represent unrealistic extremes for all the possible topologies and the timing of cladogenetic events.

### Species climatic niches

We characterized the climatic niches of each of the 134 species as follows. First, we associated climatic variable values with each of the species records (occurrences of native and introduced species in Europe). We utilized climatic information from the WorldClim database^59^ using a resolution of 2.5′ (which equals to 4 km at 30 degrees north and 3.5 km at 40 degrees north). Such a resolution was chosen to establish a reasonable compromise between computing power and spatial resolution. We then extracted values for 7 of the 18 available climatic variables (mean annual temperature, mean diurnal range, temperature seasonality, mean temperature of the wettest quarter of the year, annual precipitation, precipitation of driest month, and precipitation seasonality; see Appendix S2 for the detailed procedure we followed for climatic variable selection).

We also characterized the climatic niches of the introduced species in their native ranges, using conservative estimates of the species’ distributions. The four introduced species come from different areas. *L. humile* is native to South America, notably the Paraná River Basin. *P. megacephala* comes from Africa (Madagascar, Kenya, and Ethiopia) and the Arabian Peninsula (Yemen). *C. emeryi* also comes from Africa and is mainly found in the southernmost part of the continent. Finally, *L. neglectus* is native to the Anatolian Peninsula (Turkey). The native range of this particular species thus overlaps somewhat with our study zone (Figure S4), which is not the case for the native ranges of the three other introduced species. Species records were obtained from the four websites mentioned above as well as from a database of *L. neglectus* occurrence that is available via the CREAF website^60^. Figure S4 shows the locations where the four introduced species have been observed in Europe and their native ranges.

### Data analyses

First, we conducted a principal component analysis (PCA) from European ant data to identify 1) the main climatic variables associated with the species distributions and 2) the groups of species with similar climatic niches. The coordinates of the first two axes were used to group all species into classes: we carried out a K-means clustering analysis using the *clusplot* function (package *cluster*) in R^61^. For this analysis we used the mean climatic values, which defined the species’ optimal climatic niches. Then, we analyzed the phylogenetic signal in the species-specific means of each of the seven climatic variables. More specifically, we calculated Pagel’s ʎ and Blomberg’s K^62^ using the *phylosig* function (package *phytools*) in R. To correct for multiple testing, we applied the Holm–Bonferroni correction.

To analyze the phylogenetic signal in the optimal climatic niches of the introduced species, we ran niche overlap analyses, where niches were associated with individual climatic variables (e.g., annual precipitation niches, temperature seasonality niches). More specifically, we examined the overlap between 1) the introduced species’ climatic niches in Europe (hereafter, European climatic niches) and their climatic niches in their native ranges (hereafter, native climatic niches) and 2) the introduced species’ European climatic niches and the climatic niches of their closest relative species in Europe. The number of closest relative species was not the same for all the introduced species because we employed information from the phylogenetic tree we had constructed (Figure S3). Furthermore, in the case of *L. humile*, the ant’s closest relative species belonged to a different genus because the genus *Linepithema* is not naturally present in Europe. The identities of the closest relative species were as follows: *Linepithema humile*: *Tapinoma erraticum*, *T.* cf. *nigerrimum*, and *T. smithi*; *Pheidole megacephala*: *Pheidole pallidula*; *Cardiocondyla emeryi*: *C. batesii* and *C. elegans*; and *Lasius neglectus*: *Lasius brunneus*, *L. alienus*, *L. emarginatus*, *L. grandis*, and *L. niger*.

For the purpose to analyze niche overlap, and to predict a species’ spatial presence from its points of occurrence in its native or introduced ranges, we first calculated a convex hull (*convHull* function in the *dismo* package). We then established a 100-km wide buffer around each convex hull (*buffer* function in *raster* package) and used those two areas (hull + buffer) to define the spatial range for the values of the climatic variables. Next, we used those climatic variable values in tandem with the species occurrence data (*maxent* function in the *dismo* package) to obtain species distribution models (SDMs). We then applied cutoff values to the SDMs (see below) to obtain a two-column dataframe (to use R terminology), which included the climatic variable values for the background pixels of the area covered. These datasets were subsequently used as input for calculations performed with the *ecospat.grid.clim.dyn* function in the *ecospat* package. The cutoff value mentioned above was inferred by maximizing the true skill statistic (TSS) of the SDM determined using the combined occurrences of all the ant species.

In our first set of comparisons, we tested whether there was overlap between the European climatic niches and the native climatic niches of the four introduced species. To this end, we used a suite of functions in the *ecospat*^63^, *raster*^64^, and *dismo*^65^ packages. First, we calculated the niche overlap index (D; *ecospat.niche.overlap* in the *ecospat* package) for the two niche types (European vs. native). The value of D can range from 0 to 1, with higher values indicating greater overlap. Second, we ran a niche equivalency test (*ecospat.niche.equivalency.test* function in *ecospat* package) to statistically compare the observed overlap with the overlap between two niches built using random reallocations of observed occurrences. Third, we used the *ecospat.niche.dyn.index* function in the *ecospat* package to calculate values for following indices: niche unfilling (i.e., the proportion of occurrence densities in the native range that are associated with climatic conditions different from those associated with occurrence densities in Europe), niche expansion (i.e., the proportion of occurrence densities in Europe that are associated with climatic conditions different from those associated with occurrence densities in the native range, or 1-stability), and niche stability (i.e., the proportion of occurrence densities in Europe for which there is overlap in climatic conditions with occurrence densities in the native range)^17^. The *ecospat.niche.dyn.index* function does not allow for adjustments to occurrence densities based on the prevalence of climatic conditions within a given range. Consequently, for the sake of consistency, we did not apply that correction to any of the niche overlap calculations.

In our second set of comparisons, we tested whether there was overlap between the introduced species’ European climatic niches and the climatic niches of their closest relative species in Europe. We performed the same analyses as in the first set of comparisons. For each introduced species, the data for all its closest relative species were combined. For the niche equivalency tests, we applied the Holm–Bonferroni correction within each set of outcomes (i.e., we adjusted for the fact we were conducting 7 tests in each category, which rises from the combination of introduced species and type of overlap).

We further analyzed differences in niche overlap values using a two-way ANOVA, where the response variable was the niche overlap index (D) computed from the previous niche overlap analyses, and the explanatory variables were species (the four introduced species), comparison type (European niche vs. native niche and European niche vs. closest relative species niche), and their interactions. The replicates were the niche overlap values for each climatic variable.

Finally, we applied the same methodological framework to analyze whether there was overlap between the native climatic niches of introduced species and the climatic conditions present in Europe. To this end, we calculated the values of the niche stability index (the proportion of occurrence densities in the native range that were associated with climatic conditions also present in Europe). Climatic variable values for the full study zone were obtained using Bioclim rasters to create a comprehensive mask (Figure S1).

## Results

### Climatic niches of native and introduced species in Europe

**Figure 2:**
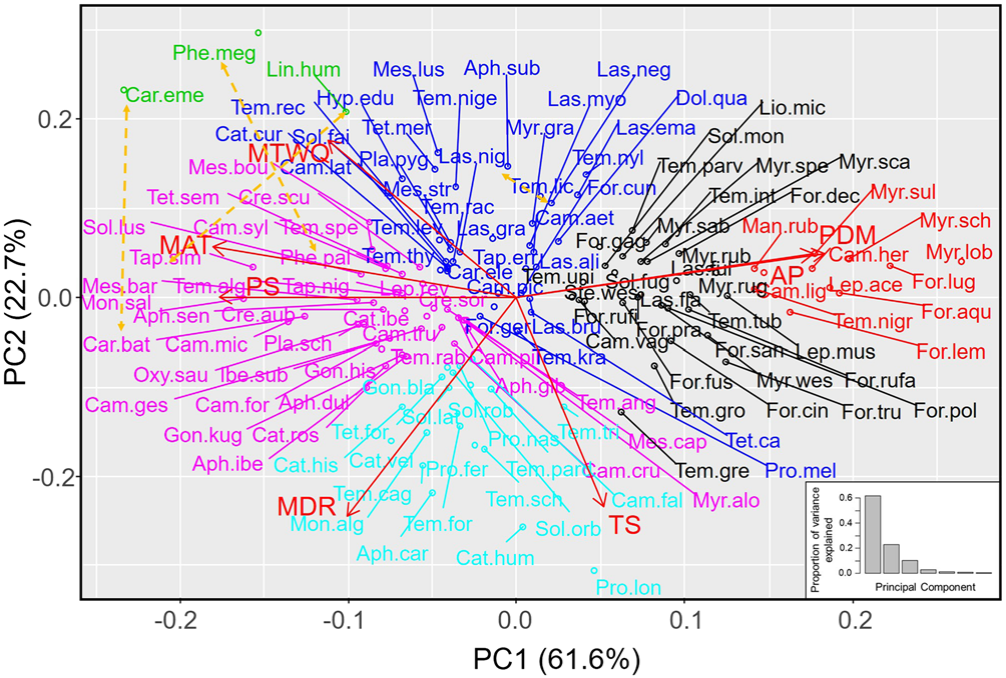
Principal components analysis (PCA) of (**a**) the 134 ant species; seven climatic variables were used to characterize the climatic niches. The existence of groups (indicated by different colors) was examined using K-means clustering analysis. Orange lines link each introduced species with its closest relative species in Europe in PCA space. See Fig. 1 for the climatic variable abbreviations. See Table S1 for the species name abbreviations.

The first two principal components of the PCA explained 84% of the total variance, highlighting the importance of climate in determining the distribution of ant species in Europe (Fig. 2, Table S3). K-means clustering analysis revealed the existence of six groups of species (Figure S5). Axis 1 explained 62% of the variance and distinguished species typically found in warm, dry areas with high precipitation seasonality from species typically found in cold areas with low precipitation seasonality. This axis clearly separated Mediterranean species (in pink in Fig. 2; largely from the genera *Messor*, *Camponotus*, *Aphaenogaster*, *Crematogaster*, *Temnothorax*, *Tapinoma*, and *Goniomma*) from boreal species (in red in Fig. 2; largely from the genera *Myrmica*, *Formica*, and *Camponotus*). There were also three groups of species (in blue, black, and light blue in Fig. 2) that were associated with milder conditions. Axis 2 explained 23% of the total variance and made these distinctions clearer: it separated species typically found in areas with highly variable temperatures (both daily and seasonally; in light blue in Fig. 2) from species typically found in areas with high temperatures in the wettest quarter of the year (in blue and black in Fig. 2). Finally, there was a group of species (in green in Fig. 2) that was clearly distinct from the other groups. This group was composed of three of the introduced species: *L. humile*, *P. megacephala*, and *C. emeryi*. These species were typically found in the warmest and driest areas that displayed both the lowest temperature seasonality and the highest precipitation seasonality of Europe. Interestingly, species from the same genera usually grouped together. Furthermore, even though the four introduced ant species belong to different and distantly related clades (Fig. 1), three of them (*L. humile*, *P. megacephala*, and *C. emeryi*) were clustered near to each other but far away from the other species within the climatic niche space represented in the PCA analysis; this distance was especially apparent along axis 2 (Fig. 2a). The fourth introduced species, *L. neglectus*, whose native range overlaps with the study zone, was located near to its closest relative species; however, it also occurred in the upper part of the graph, along axis 1 (Fig. 2).

In line with our first prediction and the PCA results, we found a strong phylogenetic signal in most of the climatic variables examined, which described the ants’ optimal climatic niches (Table 1, Fig. 1). More specifically, the signal was strong for mean annual temperature, mean diurnal range, annual precipitation, precipitation of the driest month and precipitation seasonality; it was nonexistent for temperature seasonality and for mean temperature of the wettest quarter of the year. Interestingly, these two latter variables were associated with PCA axis 2 (Table S3), which clearly differentiates three of the introduced species (*L. humile*, *P. megacephala*, and *C. emeryi;* in dark blue in Fig. 2) from the native species.

**Table 1:** Output of the phylogenetic signal tests for the climatic variables and climatic niches (Pagel’s λ and Blomberg’s K).

Climatic variable	Pagel’s λ	*P*	Blomberg’s K	*P*
Precipitation of driest month (PDM)	0.94	** < 0.0001**	0.21	0.001
Precipitation seasonality (PS)	0.93	** < 0.0001**	0.23	0.001
Mean annual temperature (MAT)	0.92	** < 0.0001**	0.23	0.001
Annual precipitation (AP)	0.91	** < 0.0001**	0.16	0.007
Mean diurnal range (MDR)	0.77	**0.0006**	0.11	0.037
Mean temperature of the wettest quarter of the year (MTWQ)	0.41	0.058	0.09	0.220
Temperature seasonality (TS)	0.20	0.034	0.10	0.196

The climatic variables are ordered from the highest to the lowest values of Pagel’s λ. In bold, significant values (*P* < 0.05) after applying the Holm–Bonferroni adjustment.

### Limited overlap in climatic niches

The results of the niche overlap analyses (Table 2) revealed that, for most of the climatic variables, introduced species did not have similar climatic niches in Europe and in their native ranges; furthermore, their European climatic niches did not resemble those of their closest relative species in Europe (Table 2). The equivalency tests showed that only *L. neglectus* and *P. megacephala* occupied equivalent annual precipitation niches (European niche vs. native niche and European niche vs. closest relative species niche, respectively). The lowest degree of niche overlap was seen for the mean temperature of the wettest quarter of the year for *L. humile*, *P. megacephala* and *C. emeryi* (European niche vs. native niche; Table 2). The niche indices computed from these niche overlap analyses indicated that the differences between the introduced species’ European niches and those of their closest relative species were mainly due to expansion rather than to unfilling (Table 2). In contrast, the differences between the introduced species’ European niches and native niches were due to both expansion and unfilling (Table 2).

**Table 2:** Niche overlap (D) and its statistical significance (*P*) based on the equivalency test.

Species	Climatic variable	European and native niches					European niches and niches of all closest relative species					Native niches and European conditions
		Overlap (D)	*P*	Unfiling	Expansion	Stability	Overlap (D)	*P*	Unfiling	Expansion	Stability	Stability
*Linepithema humile*	MAT	0.50	** < 0.0001**	0.13	0.20	0.80	0.74	** < 0.0001**	0.00	0.03	0.97	0.61
	MDR	0.37	** < 0.0001**	0.47	0.00	1.00	0.71	** < 0.0001**	0.00	0.00	1.00	0.74
	TS	0.36	** < 0.0001**	0.30	0.00	1.00	0.65	** < 0.0001**	0.00	0.00	1.00	0.41
	MTWQ	0.03	** < 0.0001**	0.95	0.60	0.40	0.74	** < 0.0001**	0.00	0.01	0.99	0.06
	AP	0.11	** < 0.0001**	0.64	0.00	1.00	0.87	** < 0.0001**	0.00	0.00	1.00	0.52
	PDM	0.16	** < 0.0001**	0.62	0.09	0.91	0.85	** < 0.0001**	0.00	0.02	0.98	0.57
	PS	0.44	** < 0.0001**	0.36	0.00	1.00	0.80	** < 0.0001**	0.00	0.00	1.00	0.82
*Pheidole megacephala*	MAT	0.37	** < 0.0001**	0.06	0.45	0.55	0.58	**0.01**	0.00	0.10	0.90	0.75
	MDR	0.39	** < 0.0001**	0.01	0.38	0.62	0.51	** < 0.0001**	0.00	0.23	0.77	0.98
	TS	0.40	** < 0.0001**	0.46	0.00	1.00	0.45	** < 0.0001**	0.00	0.00	1.00	0.07
	MTWQ	0.16	** < 0.0001**	0.33	0.70	0.30	0.67	**0.01**	0.00	0.21	0.79	0.34
	AP	0.22	** < 0.0001**	0.00	0.65	0.35	0.75	0.29	0.00	0.05	0.95	1.00
	PDM	0.28	** < 0.0001**	0.00	0.58	0.42	0.40	** < 0.0001**	0.00	0.25	0.75	1.00
	PS	0.48	** < 0.0001**	0.00	0.26	0.74	0.43	** < 0.0001**	0.00	0.00	1.00	1.00
*Cardiocondyla emeryi*	MAT	0.59	**0.02**	0.00	0.37	0.63	0.48	**0.01**	0.00	0.22	0.78	0.89
	MDR	0.51	** < 0.0001**	0.00	0.13	0.87	0.61	**0.02**	0.06	0.00	1.00	1.00
	TS	0.60	** < 0.0001**	0.08	0.00	1.00	0.46	**0.01**	0.00	0.01	0.99	0.65
	MTWQ	0.12	** < 0.0001**	0.19	0.83	0.17	0.63	**0.03**	0.00	0.28	0.72	0.40
	AP	0.21	** < 0.0001**	0.00	0.60	0.40	0.51	** < 0.0001**	0.00	0.03	0.97	1.00
	PDM	0.12	** < 0.0001**	0.00	0.85	0.15	0.23	** < 0.0001**	0.00	0.71	0.29	1.00
	PS	0.47	** < 0.0001**	0.00	0.43	0.57	0.34	** < 0.0001**	0.00	0.53	0.47	1.00
*Lasius neglectus*	MAT	0.64	** < 0.0001**	0.00	0.16	0.84	0.76	** < 0.0001**	0.00	0.05	0.95	0.99
	MDR	0.66	**0.01**	0.01	0.24	0.76	0.74	** < 0.0001**	0.00	0.08	0.92	0.99
	TS	0.54	** < 0.0001**	0.00	0.06	0.94	0.75	** < 0.0001**	0.00	0.03	0.97	0.92
	MTWQ	0.61	** < 0.0001**	0.07	0.01	0.99	0.79	** < 0.0001**	0.00	0.00	1.00	0.96
	AP	0.72	0.07	0.00	0.00	1.00	0.55	** < 0.0001**	0.00	0.10	0.90	0.94
	PDM	0.31	** < 0.0001**	0.37	0.00	1.00	0.63	** < 0.0001**	0.00	0.01	0.99	0.89
	PS	0.29	** < 0.0001**	0.00	0.19	0.81	0.59	** < 0.0001**	0.00	0.03	0.97	1.00

For each climatic variable and introduced ant species, there were two types of comparisons: (1) a comparison of the overlap between the introduced species’ European and native climatic niches and (2) a comparison of the overlap between the introduced species’ European climatic niches and those of their closest relative species in Europe (all closest relative species combined). Niche unfilling, expansion, and stability indices were also determined for each comparison. Furthermore, for each climatic variable and introduced ant species, an additional version of the niche stability index was calculated: it quantified how the native climatic niches of introduced species fit with climatic conditions in Europe. Abbreviations: *MAT* mean annual temperature; *MDR* mean diurnal range (mean of monthly [max temp − min temp]); *TS* temperature seasonality; *MTWQ* mean temperature of wettest quarter of the year; *AP* annual precipitation; *PDM* precipitation of driest month; *PS* precipitation seasonality. In bold, significant values (*P* < 0.05) after applying the Holm–Bonferroni adjustment.

**Figure 3:**
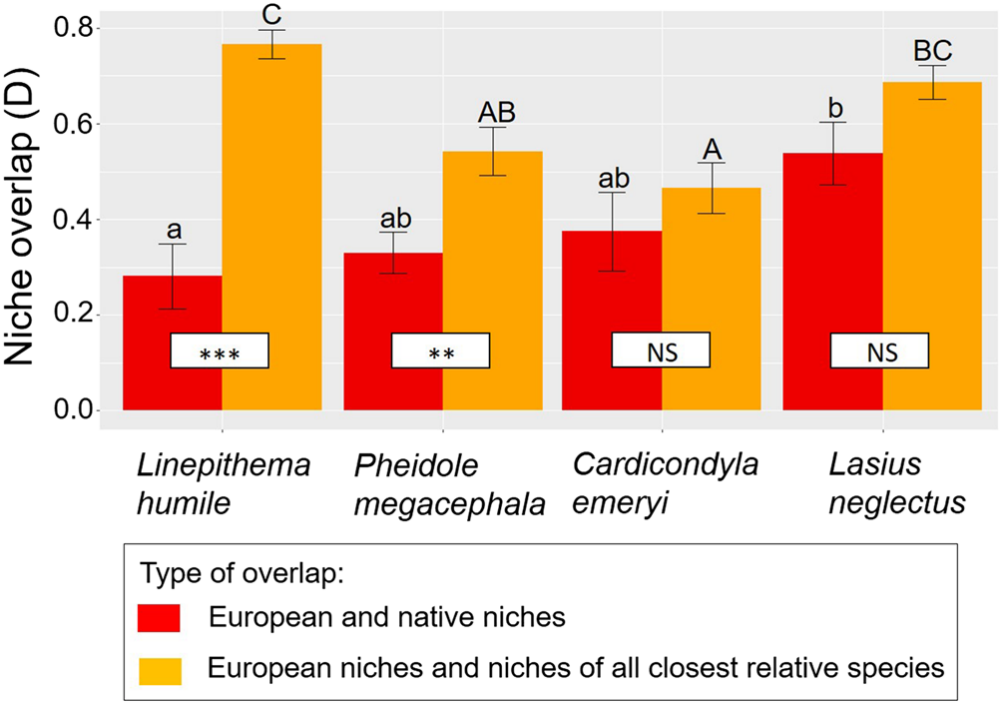
Niche overlap (D) values between European and native niches (red bars) and between European niches and niches of all closest relative species (orange bars) for each introduced ant species. The results of the two-way ANOVA test are included in the figure. Thus, the significance of differences (*NS*, no significant; **, *p* < 0.001; ***, *P* < 0.0001) between type of overlap (between European and native niches and between European niches and niches of all closest relative species) for each species is shown at the basis of the bars. The differences among species within each type of overlap were tested with post-hoc Tukey tests comparing least square means and shown in the upper part of the bars: lower case and capital letters depict significant differences among species in overlap (D) values between European and native niches (red bars) and between European niches and niches of all closest relative species (orange bars), respectively.

A further ANOVA testing for differences in niche overlap values (computed from the previous niche overlap analyses) between species (the four introduced species) and comparison type (European niche vs. native niche and European niche vs. closest relative species niche) revealed that niche overlap values were affected by the interaction between species and comparison type (two-way ANOVA; F_3,48_ = 4.9, *P* = 0.005). On average, there was an intermediate degree of niche overlap and thus no differences among the introduced species (Fig. 3). For *L. humile* and *P. megacephala*, the overlap between their European niches and the niches of their closest relative species was greater than the overlap between their European niches and their native niches; no such difference was seen for *C. emeryi* and *L. neglectus* (Fig. 3). *L. neglectus* had the greatest overlap between its European niche and its native niche; *L. humile* had the smallest (Fig. 3). *L. humile* had the greatest overlap between its European niche and the niches of its closest relative species, and *C. emeryi* had the smallest (Fig. 3).

### Do introduced species have access to the same climatic niches in Europe as in their native ranges?

We also analyzed the degree of overlap between the introduced species’ native niches and the niches available in the overall study zone (Table 2) using the niche stability index. In the case of *L. humile*, niche stability values were intermediate (0.40–0.75) for most climatic variables; however, for mean temperature of the wettest quarter of the year and precipitation seasonality, they were very low (< 0.10) and high (> 0.80), respectively. For *P. megacephala* and *C. emeryi*, niche stability values were high, except in the case of temperature seasonality and mean temperature of the wettest quarter of the year (Table 2). These results indicate that certain climatic niches or climatic axes occupied by these species in their native ranges do not correspond much to climatic conditions in the study zone. In contrast, for *L. neglectus*, stability values were very high for all the climatic variables (Table 2), suggesting that this species could exploit climatic niches in Europe that are available in its native range.

## Discussion

### Phylogeny constrains the climatic niches of native European ants

According to our first prediction, we found that closely related species have very similar optimal climatic niches, which suggests that ant species distributions in Europe are constrained by evolutionary history. Our results support the prediction that poikilotherms should exhibit strong phylogenetic signals in thermal preferences. Indeed, our results agree with those found in other insects (e.g., *Drosophila*^3^) and poikilotherms (e.g., amphibians^7^; salamanders^18^), and contrast with findings in endothermic taxa such as birds^19^ and mammals^6,9,22–24^, which are expected to show weaker phylogenetic patterns.

A few previous studies have also focused on ants but have obtained contrasting results. For instance, Lessard et al.^42^ reported that the broad-scale climatic niches of closely related ant species in North America were more similar to each other than they were to those of more phylogenetically distant species. Pie^10^ found a strong phylogenetic signal in ant climatic niches in a genus-level analysis carried out at a global scale. In contrast, Lucky et al.^43^ found that related ant genera from tropical, subtropical, and temperate areas were not more likely than unrelated genera to occupy similar biomes. Similarly, Economo et al.^44^ showed that the climatic niches of *Pheidole* species distributed across the globe were highly labile and little influenced by relatedness. The scale of analysis may be at play in these contrasting results^5^, given that signals should be more evident at broad phylogenetic and spatial scales. Regardless, our findings are clear: there is evidence of phylogenetic constraints in the evolution of the optimal climatic niches of native European ant species.

Taking a closer look, the differentiation between Mediterranean and boreal ant species represents an important facet of these results. Indeed, our research supports the idea that warmer regions are composed of more phylogenetically diverse ant lineages than are colder regions, which are more phylogenetically homogeneous^10^. Here, species-poor boreal ant communities were composed of a limited number of species adapted to low temperatures and high precipitation that mainly belonged to the genera *Formica* (*rufa* group), *Myrmica*, and *Camponotus*. Meanwhile, the Mediterranean communities were composed of many diverse species adapted to high temperatures and low, highly seasonal precipitation that belonged to the genera *Aphaenogaster*, *Goniomma*, *Cataglyphis*, *Tapinoma, Crematogaster*, and *Camponotus*. Our findings also support the idea that conservatism in climatic niches helps establish range limits, thereby creating biogeographical patterns of distribution and species richness^66^.

### What about the climatic niches of introduced species in Europe?

In contrast to our second prediction, phylogeny does not constrain climatic niches of introduced ant species. The four introduced ants represent the three most species-rich subfamilies of ants—Dolichoderinae, Formicinae, and Myrmicinae—which fits with the phylogenetic diversity exhibited by invasive ants that has been noted elsewhere^41^ (see also Figure S3). Since the climatic niches of native European ants are phylogenetically constrained, it could be hypothesized that introduced species should have the same climatic niches in their native and introduced ranges or that their climatic niches in their introduced ranges should resemble those of their closest relative species. However, we found strong evidence that this is not the case.

We observed that three of the four introduced species (*L. humile*, *P. megacephala*, and *C. emery*) occupied similar optimal climatic niches despite being distantly related. The fourth species, *L. neglectus*, had a slightly different climatic niche, very likely because its native range overlaps with the study zone. There are different mechanistic explanations for this pattern. First, introduced species may exploit novel habitats that are not being used by native species (e.g., human-altered environments^67,68^). Second, introduced species may have similar life-history traits to one another^69^. Third, they could have large colonies of small workers, a trait that could serve to buffer the effects of harsher European climates, given that they are coming from milder conditions in their native, tropical ranges^70,71^. In fact, the two climatic variables that seem to be the most important in distinguishing the optimal climatic niches of introduced species in Europe from the climatic niches of native European species—temperature seasonality and mean temperature of the wettest quarter of the year—do not display a phylogenetic signal. According to various studies, the different axes of species climatic niches might be shaped by different dynamics^10,22,72^. Consequently, phylogenetic constraints might have played an important role in the broader evolution of climatic niches, generating such distinctions as those between the Mediterranean and boreal ant communities, but could be less influential at smaller spatial scales^10^.

We also found that the optimal climatic niches of introduced species in Europe, with the exception of *L. neglectus*, were very different from those of the most common native European ant species. Two hypotheses could help explain these results. First, the vacant niche hypothesis states that successful invaders can use vacant niches, especially if they are novel. Second, the limiting similarity hypothesis states that successful invaders are functionally distinct from species in the recipient community—they thus encounter minimal competition and can fill empty niches^25,73^. Both predict there should be dramatic trait/phylogenetic overdispersion, which we saw in the broad range of climatic niches that emerged when all European ants were considered. We also observed that, like certain native species, the introduced species tended to occur in warm, dry areas. The difference was that when these areas were occupied by introduced species, the areas were also more likely to have low temperature variation (either daily or seasonally) and very high temperatures in the wettest quarter of the year. These are key climatic characteristics in the tropical and subtropical regions from whence these species originate. However, these features might be too harsh for most European natives, limiting their numbers in such habitats^74^.

Finally, there was no overlap between the European and native climatic niches of introduced species nor was there overlap between the European climatic niches of introduced species and the climatic niches of their closest relative species in Europe. This result contrasts with the predictions of the phylogenetic signal hypothesis^17^, namely that introduced species should have similar climatic niches in their introduced and native ranges (or that their introduced niches should resemble those of their closest relative species). In general, niche overlap values were intermediate and did not differ among introduced species (see Fig. 3). We observed that, for *L. humile* and *P. megacephala*, there was more overlap between their European climatic niches and those of their closest relative species in Europe than there was between their European and native climatic niches. This suggests that they might have a genetic predisposition for occupying climatic niches similar to those occupied by their closest relative species^17^. That said, introduced species a) did not occupy a large proportion of their native climatic niches in Europe (unfilling), b) expanded into new niches in Europe that they did not occupy in their native ranges (expansion), and c) expanded into new niches in Europe that were not occupied by their closest relative species (expansion). We feel that the first result (a) reflects that climatic conditions in Europe offer niches that are very different from those in the introduced species’ native ranges. Indeed, this explanation seems far more likely than the explanation that introduced ant species are avoiding such climatic conditions or are prevented from colonizing them. These findings can be explained by evoking climatic niche conservatism^17^ in combination with the operation of adaptive evolution during invasion^33,75^.

Taken together, these results indicate that, in Europe, the climatic niches of introduced ant species do not appear to be phylogenetically constrained. Instead, introduced species appear to occupy climatic niches that are restricted or marginally used by native ants, which fits with the predictions of the empty niche hypothesis. This pattern was true for at least some climatic variables and especially for the optimal climatic niche. The situation was different for *L*. *neglectus*, which naturally occurs on the Anatolian Peninsula. Its optimal climatic niche in Europe did not resemble those of the other three introduced ant species. Instead, *L*. *neglectus* bore a greater resemblance to the native ant species, including its closest relative species. This fact could suggest that, in addition to relatedness, biogeographical origin and colonization abilities might play a role in the evolution of climatic niches. However, the results for *L. neglectus* do not put into question our general conclusions because the full climatic niche of this species in Europe does not overlap with its climatic niche in its native range nor does it fully correspond with the climatic niches of its closest relative species.

### Implications for biodiversity conservation and management in Europe

While invasive ant species have been shown to negatively affect ant diversity^27,41^, as well as the diversity of other animals and plants^41,76^, their impacts could be limited if they occupy less used, more extreme, marginal, or restricted climatic niches and/or occur in communities with a low diversity of functionally distinct species. As a result, competitive exclusion could be avoided, as predicted by niche theory or the limiting similarity hypothesis^77,78^, and negative impacts on ant communities and the ecosystem could be reduced. Identifying and characterizing invasive species is essential when prioritizing and managing invasions, as made clear in the Aichi targets of the Convention of Biological Diversity (Strategic Plan 2020^79^). Here, we suggest that governmental efforts devoted to stopping the arrival and spread of introduced species in Europe should invest more resources in protecting environments where conditions are a closer match for the climatic niches of targeted invaders.

## Supplementary information

Supplementary information.

## Data Availability

The data forming the basis for our results will be archived in Dryad.
