# Supplementary material for: Introduced ant species occupy empty climatic niches in Europe
Source: Sci Rep. 2021 Feb 8;11:3280. doi: 10.1038/s41598-021-82982-y (PMC7870827; doi:10.1038/s41598-021-82982-y)
Supplement: Supplementary file 1 — Supplementary information. [file 41598_2021_82982_MOESM1_ESM.docx]

SUPPLEMENTARY INFORMATION (SI)

*Introduced ant species occupy empty climatic niches in Europe*

Xavier Arnan, Elena Angulo, Raphael Boulay, Roberto Molowny-Horas, Xim Cerdá, and Javier Retana

**Table S1.** Species included in this study (species-specific code, subfamily, and full species name) and their number of observations. In bold, introduced species.

**Table S2.** Ant species found in our study area that have been introduced out of its native range in the world, following the compilation by Bertelsmeier et al. (2017) and Fournier et al. (2018). We describe the native range following different sources (Seifert 2018, Bernard 1968, AntWeb and AntWiki) and the status in Europe (Status in Europe): whether the species is native in Europe (Native), introduced (Introduced), has been found introduced somewhere in Europe (i.e. N. Europe), or their status is dubious (?). Ant species are ordered in alphabetic order and in three categories: (A) the native ant species in our database following the distribution of locations used (see also Figure S2), (B) the introduced ant species, and (C) the ones that have been removed due to uncertainty about the origin and history. The column Ref contains other relevant references than the ones already stated (complete reference list included below).

**Table S3.** Outputs from the principal component analyses (PCA) of the seven bioclimatic variables for all ant species. The variance explained by each of the first two axes and the contributions of the original variables to these axes are given. The values correspond to the squared correlation coefficients.

**Figure S1.** Map of the study zone for which species records were obtained. Map has been created by combining species records and the object wrld_simple in the *maptools* package in R (R Core Team 2016, URL <http://www.R-project.org/>).

**Figure S2.** Maps of species records of the five ant species found in our study area that have been introduced out of its native range somewhere in Europe (i.e. N. Europe). We show the locations used, confirming that any of the introduced locations is comprised in our dataset. More information in Table S2. Maps have been created by combining species records and the object wrld_simple in the *maptools* package in R (R Core Team 2016, URL <http://www.R-project.org/>).

**Figure S3.** Ant phylogeny used in this study.

**Figure S4.** Species records for the four introduced species and their phylogenetically closest relative species. Maps have been created by combining species records and the object wrld_simple in the *maptools* package in R (R Core Team 2016, URL <http://www.R-project.org/>).

**Figure S5.** Plot of within-groups sum of squares against number of clusters using the k-means approach in order to choose the number of clusters for the PCA.

**Appendix S1.** List of references used to build the working phylogeny for the 134 European ant species examined in this study.

**Appendix S2.** Selection of predictor climate variables.

**Table S1.** Species included in this study (species-specific code, subfamily, and full species name) and their number of observations (N). In bold, introduced species.

| Code | Subfamily | Species | N |
| --- | --- | --- | --- |
| Dol.qua | Dolichoderinae | *Dolichoderus quadripunctatus* | 37 |
| **Lin.hum** | **Dolichoderinae** | ***Linepithema humile*** | **335** |
| Lio.mic | Dolichoderinae | *Liometopum microcephalum* | 16 |
| Tap.err | Dolichoderinae | *Tapinoma erraticum* | 306 |
| Tap.nig | Dolichoderinae | *Tapinoma* cf. *nigerrimum* | 453 |
| Tap.sim | Dolichoderinae | *Tapinoma simrothi* | 162 |
| Cam.aet | Formicinae | *Camponotus aethiops* | 250 |
| Cam.cru | Formicinae | *Camponotus cruentatus* | 337 |
| Cam.fal | Formicinae | *Camponotus fallax* | 58 |
| Cam.for | Formicinae | *Camponotus foreli* | 142 |
| Cam.ges | Formicinae | *Camponotus gestroi* | 41 |
| Cam.her | Formicinae | *Camponotus herculeanus* | 35 |
| Cam.lat | Formicinae | *Camponotus lateralis* | 339 |
| Cam.lig | Formicinae | *Camponotus ligniperdus* | 59 |
| Cam.mic | Formicinae | *Camponotus micans* | 152 |
| Cam.pic | Formicinae | *Camponotus piceus* | 194 |
| Cam.pil | Formicinae | *Camponotus pilicornis* | 190 |
| Cam.syl | Formicinae | *Camponotus sylvaticus* | 221 |
| Cam.tru | Formicinae | *Camponotus truncatus* | 139 |
| Cam.vag | Formicinae | *Camponotus vagus* | 100 |
| Cat.cur | Formicinae | *Cataglyphis cursor* | 27 |
| Cat.his | Formicinae | *Cataglyphis hispanica* | 70 |
| Cat.hum | Formicinae | *Cataglyphis humeya* | 10 |
| Cat.ibe | Formicinae | *Cataglyphis iberica* | 120 |
| Cat.ros | Formicinae | *Cataglyphis rosenhaueri* | 40 |
| Cat.vel | Formicinae | *Cataglyphis velox* | 82 |
| For.aqu | Formicinae | *Formica aquilonia* | 190 |
| For.cin | Formicinae | *Formica cinerea* | 72 |
| For.cun | Formicinae | *Formica cunicularia* | 153 |
| For.dec | Formicinae | *Formica decipiens* | 40 |
| For.fus | Formicinae | *Formica fusca* | 187 |
| For.gag | Formicinae | *Formica gagates* | 44 |
| For.ger | Formicinae | *Formica gerardi* | 88 |
| For.lem | Formicinae | *Formica lemani* | 70 |
| For.lug | Formicinae | *Formica lugubris* | 30 |
| For.pol | Formicinae | *Formica polyctena* | 57 |
| For.pra | Formicinae | *Formica pratensis* | 154 |
| For.rufa | Formicinae | *Formica rufa* | 73 |
| For.rufi | Formicinae | *Formica rufibarbis* | 186 |
| For.san | Formicinae | *Formica sanguinea* | 112 |
| For.sub | Formicinae | *Formica subrufa* | 186 |
| For.tru | Formicinae | *Formica truncorum* | 40 |
| Las.ali | Formicinae | *Lasius alienus* | 314 |
| Las.bru | Formicinae | *Lasius brunneus* | 75 |
| Las.ema | Formicinae | *Lasius emarginatus* | 136 |
| Las.fla | Formicinae | *Lasius flavus* | 208 |
| Las.ful | Formicinae | *Lasius fuliginosus* | 73 |
| Las.gra | Formicinae | *Lasius grandis* | 106 |
| Las.myo | Formicinae | *Lasius myops* | 75 |
| **Las.neg** | **Formicinae** | ***Lasius neglectus*** | **154** |
| Las.nig | Formicinae | *Lasius niger* | 487 |
| Pla.pyg | Formicinae | *Plagiolepis pygmaea* | 495 |
| Pla.sch | Formicinae | *Plagiolepis schmitzii* | 329 |
| Pro.fer | Formicinae | *Proformica ferreri* | 20 |
| Pro.lon | Formicinae | *Proformica longiseta* | 13 |
| Pro.nas | Formicinae | *Proformica nasuta* | 23 |
| Lep.rev | Leptanillinae | *Leptanilla revelieri* | 21 |
| Aph.car | Myrmicinae | *Aphaenogaster cardenai* | 13 |
| Aph.dul | Myrmicinae | *Aphaenogaster dulcineae* | 53 |
| Aph.gib | Myrmicinae | *Aphaenogaster gibbosa* | 282 |
| Aph.ibe | Myrmicinae | *Aphaenogaster iberica* | 262 |
| Aph.sen | Myrmicinae | *Aphaenogaster senilis* | 202 |
| Aph.sub | Myrmicinae | *Aphaenogaster subterranea* | 134 |
| Car.bat | Myrmicinae | *Cardiocondyla batesii* | 62 |
| Car.ele | Myrmicinae | *Cardiocondyla elegans* | 31 |
| **Car.eme** | **Myrmicinae** | ***Cardiocondyla emeryi*** | **14** |
| Cre.aub | Myrmicinae | *Crematogaster auberti* | 370 |
| Cre.scu | Myrmicinae | *Crematogaster scutellaris* | 542 |
| Cre.sor | Myrmicinae | *Crematogaster sordidula* | 221 |
| Gon.bla | Myrmicinae | *Goniomma blanci* | 24 |
| Gon.his | Myrmicinae | *Goniomma hispanicum* | 60 |
| Gon.kug | Myrmicinae | *Goniomma kugleri* | 14 |
| Lep.ace | Myrmicinae | *Leptothorax acervorum* | 44 |
| Lep.mus | Myrmicinae | *Leptothorax muscorum* | 28 |
| Man.rub | Myrmicinae | *Manica rubida* | 16 |
| Mes.bar | Myrmicinae | *Messor barbarus* | 375 |
| Mes.bou | Myrmicinae | *Messor bouvieri* | 330 |
| Mes.cap | Myrmicinae | *Messor capitatus* | 265 |
| Mes.lus | Myrmicinae | *Messor lusitanicus* | 19 |
| Mes.str | Myrmicinae | *Messor structor* | 236 |
| Mon.alg | Myrmicinae | *Monomorium algiricum* | 15 |
| Mon.sal | Myrmicinae | *Monomorium salomonis* | 195 |
| Myr.gra | Myrmicinae | *Myrmecina graminicola* | 160 |
| Myr.alo | Myrmicinae | *Myrmica aloba* | 107 |
| Myr.lob | Myrmicinae | *Myrmica lobulicornis* | 19 |
| Myr.rub | Myrmicinae | *Myrmica rubra* | 56 |
| Myr.rug | Myrmicinae | *Myrmica ruginodis* | 73 |
| Myr.sab | Myrmicinae | *Myrmica sabuleti* | 102 |
| Myr.sca | Myrmicinae | *Myrmica scabrinodis* | 103 |
| Myr.sch | Myrmicinae | *Myrmica schencki* | 23 |
| Myr.spe | Myrmicinae | *Myrmica specioides* | 30 |
| Myr.sul | Myrmicinae | *Myrmica sulcinodis* | 76 |
| Myr.wes | Myrmicinae | *Myrmica wesmaeli* | 43 |
| Oxy.sau | Myrmicinae | *Oxyopomyrmex saulcyi* | 31 |
| **Phe.meg** | **Myrmicinae** | ***Pheidole megacephala*** | **16** |
| Phe.pal | Myrmicinae | *Pheidole pallidula* | 758 |
| Sol.fai | Myrmicinae | *Solenopsis fairchildi* | 36 |
| Sol.fug | Myrmicinae | *Solenopsis fugax* | 69 |
| Sol.lat | Myrmicinae | *Solenopsis latro* | 41 |
| Sol.lus | Myrmicinae | *Solenopsis lusitanica* | 13 |
| Sol.mon | Myrmicinae | *Solenopsis monticola* | 17 |
| Sol.orb | Myrmicinae | *Solenopsis orbula* | 18 |
| Sol.rob | Myrmicinae | *Solenopsis robusta* | 15 |
| Ste.wes | Myrmicinae | *Stenamma westwoodi* | 65 |
| Tem.alg | Myrmicinae | *Temnothorax algiricus* | 39 |
| Tem.ang | Myrmicinae | *Temnothorax angustulus* | 37 |
| Tem.cag | Myrmicinae | *Temnothorax cagnianti* | 14 |
| Tem.for | Myrmicinae | *Temnothorax formosus* | 53 |
| Tem.gre | Myrmicinae | *Temnothorax gredosi* | 16 |
| Tem.gro | Myrmicinae | *Temnothorax grouvellei* | 22 |
| Tem.int | Myrmicinae | *Temnothorax interruptus* | 29 |
| Tem.kra | Myrmicinae | *Temnothorax kraussei* | 23 |
| Tem.lev | Myrmicinae | *Temnothorax leviceps* | 12 |
| Tem.lic | Myrmicinae | *Temnothorax lichtensteini* | 54 |
| Tem.nige | Myrmicinae | *Temnothorax niger* | 32 |
| Tem.nigr | Myrmicinae | *Temnothorax nigriceps* | 11 |
| Tem.nyl | Myrmicinae | *Temnothorax nylanderi* | 67 |
| Tem.pard | Myrmicinae | *Temnothorax pardoi* | 44 |
| Tem.parv | Myrmicinae | *Temnothorax parvulus* | 26 |
| Tem.rab | Myrmicinae | *Temnothorax rabaudi* | 41 |
| Tem.rac | Myrmicinae | *Temnothorax racovitzai* | 96 |
| Tem.rec | Myrmicinae | *Temnothorax recedens* | 224 |
| Tem.sch | Myrmicinae | *Temnothorax schaufussi* | 15 |
| Tem.spe | Myrmicinae | *Temnothorax specularis* | 95 |
| Tem.tri | Myrmicinae | *Temnothorax tristis* | 32 |
| Tem.tub | Myrmicinae | *Temnothorax tuberum* | 22 |
| Tem.thy | Myrmicinae | *Temnothorax thyndalei* | 74 |
| Tem.uni | Myrmicinae | *Temnothorax unifasciatus* | 110 |
| Tet.cae | Myrmicinae | *Tetramorium caespitum* | 517 |
| Tet.for | Myrmicinae | *Tetramorium forte* | 248 |
| Tet.mer | Myrmicinae | *Tetramorium meridionale* | 47 |
| Tet.sem | Myrmicinae | *Tetramorium semilaeve* | 479 |
| Hyp.edu | Ponerinae | *Hypoponera eduardi* | 135 |
| Pro.mel | Proceratiinae | *Proceratium melinum* | 23 |

**Table S2.** Ant species found in our study area that have been introduced out of its native range in the world, following the compilation by Bertelsmeier et al. (2017) and Fournier et al. (2018). We describe the native range following different sources (Seifert 2018, Bernard 1968, AntWeb and AntWiki) and the status in Europe (Status in Europe): whether the species is native in Europe (Native), introduced (Introduced), has been found introduced somewhere in Europe (i.e. N. Europe), or their status is dubious (?). Ant species are ordered in alphabetic order and in three categories: (A) the native ant species in our database following the distribution of locations used (see also Figure S2), (B) the introduced ant species, and (C) the ones that have been removed due to uncertainty about the origin and history. The column Ref contains other relevant references than the ones already stated (complete reference list included below).

|  | **Ant species** | **Native range** | | | **Status in Europe** | **Ref** |
| --- | --- | --- | --- | --- | --- | --- |
|  |  | **Seifert 2018** | **Bernard 1968** | **AntWeb & AntWiki** |  |  |
|  | | |  |  |  |  |
| **A. Ant species considered native in our database:** | | |  |  |  |  |
|  | *Crematogaster scutellaris* | West mediterranean | Mediterranean | Paleartic - Mediterranean Europe | N Europe | 1,2 |
|  | *Crematogaster sordidula* | Holomediterranean | Mediterranean, N & E Africa | Paleartic - Mediterranean Europe | N Europe |  |
|  | *Camponotus vagus* | European - W Siberian | Mediterranean, Europe and Asia | Paleartic - Europe & Asia, N Africa | N Europe | 3-6 |
|  | *Camponotus fallax* | Eurosiberian | Central & S Europe | Paleartic - Central & S Europe | Native |  |
|  | *Camponotus herculeanus* | Panpaleartic | Central & N Europa, Asia | Paleartic – Neartic | Native |  |
|  | *Camponotus lateralis* | Eurocaucasian-Mediterranean | Mediterranean | Paleartic - Mediterranean | Native |  |
|  | *Dolichoderus quadripunctatus* | Westpaleartic | Central & S Europe, West Asia | Paleartic - W Europe | Native |  |
|  | *Formica fusca* | Eurocaucasian | Europe & N Africa | Paleartic – Neartic | Native |  |
|  | *Formica rufa* | European | Europe & Asia | Paleartic - Europe | Native |  |
|  | *Hypoponera eduardi* | Holomediterranean | Mediterranean | Paleartic | N Europe |  |
|  | *Lasius alienus* | Eurosiberian | Europe & N America | Paleartic - Europe & Asia | Native |  |
|  | *Lasius emarginatus* | European | Central & W Europe | Paleartic | Native |  |
|  | *Lasius flavus* | Panpaleartic | Holartic, Mediterranean | Paleartic | Native |  |
|  | *Lasius fuliginosus* | Paleartic | Europe | Paleartic | Native |  |
|  | *Lasius niger* | Eurosiberian | Europe & Asia, Mediterranean | Paleartic - Europe | Native |  |
|  | *Leptothorax muscorum* | Transpaleartic | North Europe & Asia | Paleartic | Native |  |
|  | *Monomorium salomonis* |  | S Mediterranean & N Africa | S Europe & N. Africa | Native |  |
|  | *Myrmica rubra* | Eurosiberian | Europe & Asia | Paleartic - Europe | Native | 7 |
|  | *Myrmica ruginodis* | Panpaleartic | Europe & Asia | Paleartic | Native | 7 |
|  | *Solenopsis fugax* | W Paleartic, Mediterranean | Europe & Asia, N Africa | Paleartic | Native |  |
|  | *Tapinoma cf. nigerrimum* | Central & W Mediterranean | Mediterranean & Asia | Paleartic - Mediterranean | N Europe | 8 |
|  | *Tapinoma erraticum* | European | Europe & Asia | Paleartic | Native | 9 |
|  | *Tetramorium caespitum* | European | Europe & Asia | Paleartic | Native |  |
|  |  |  |  |  |  |  |
| **B. Ant species introduced in Europe:** | | |  |  |  |  |
|  | *Cardiocondyla emeryi* |  |  | Africa |  | 10-11 |
|  | *Lasius neglectus* | Asia Minor |  | Paleartic - West Asia | Introduced |  |
|  | *Linepithema humile* | Paraná basin (S America) |  | South America - Paraná River Bassin | Introduced |  |
|  | *Pheidole megacephala* |  |  | Afrotropical - Malagasy | Introduced |  |
|  |  |  |  |  |  |  |
| **C. Ant species with doubious origin and history:** | | |  |  |  |  |
|  | *Cardiocondyla mauritanica* |  | Tunisia & Sahara | E Paleartic & W Asia (AntWeb)  E Asia & N Africa (AntWiki) | ? | 11-12-13 |
|  | *Hypoponera punctatissima* | N & W Europe | W Europe (France, Italy) | Afrotropical - Africa or Central Asia | ? | 14-15 |
|  |  |  |  |  |  |  |

References cited:

1. Sellenschlo, U. (1993). "Cremastogaster <sic> scutellaris (Oliv.) (Hym., Myrmicidae) nach Norddeutschland eingeschleppt." Anz. Schadlingskd. Pflanzenschutz Umweltschutz 66: 105-107.
2. Vierbergen, G. (1994). Hymenoptera / Formicidae / Crematogaster scutellaris in The Netherlands, Verslagen en Mededelingen (Annual Report 1993, Plant Protection Service, Wageningen, The Netherlands) 173: 51-52.
3. Dekoninck, W. and P. Pauly (2002). "Camponotus vagus Scopoli 1763 (Hymenoptera: Formicidae) a new ant species for Belgium?" Bull. Ann. Soc. R. Entomol. Belg. 138(1-6): 29-30.
4. Kvamme, T. and O. J. Lønnve (2008). "Camponotus vagus (Scopoli, 1763) (Hymenoptera, Formicidae) in Norway." Norw. J. Entomol. 55(1): 105-108.
5. Lomholdt, O. (1988). "Myrerne Oecophylla smaragdina (Fabricius, 1775) og Camponotus vagus (Scopoli, 1763) fundet i Danmark." Entomol. Medd. 56(2): 72.
6. Zięba, P. and M. Chorągwicki (2009). "Nowe stanowiska Camponotus vagus (Scopoli, 1763) (Formicidae) na Nizinie Sandomierskiej." Acta Entomol. Siles. 17: 82-83.
7. Leppänen, J., et al. (2013). "Comparative phylogeography of the ants Myrmica ruginodis and Myrmica rubra." J. Biogeogr. 40(3): 479-491
8. Seifert, B. (2017). "Four species within the supercolonial ants of the Tapinoma nigerrimum complex revealed by integrative taxonomy (Hymenoptera: Formicidae)" Myrmecological News, 24:123-144
9. Seifert, B. (1984). "A method for differentiation of the female castes of Tapinoma ambiguum Emery and Tapinoma erraticum (Latr.) and remarks on their distribution in Europe north of the Mediterranean region." Faun. Abh. (Dres.) 11(11): 151-155.
10. Wetterer, J. K. (2012). "Worldwide spread of Emery's sneaking ant, Cardiocondyla emeryi (Hymenoptera: Formicidae)." Myrmecol. News 17: 13-20.
11. Heinze, J., et al. (2006). "Stealthy invaders: the biology of Cardiocondyla tramp ants." Insect. Soc. 53(1): 1-7.
12. Wetterer, J. K. (2012). "Worldwide spread of the moorish sneaking ant, Cardiocondyla mauritanica (Hymenoptera: Formicidae)." Sociobiology 59(3): 985-997.
13. Bernard, F. (1956). "Révision des fourmis paléarctiques du genre Cardiocondyla Emery." Bull. Soc. Hist. Nat. Afr. Nord 47: 299-306.
14. Delabie, J. H. C. and F. Blard (2002). "The tramp ant Hypoponera punctatissima (Roger) (Hymenoptera: Formicidae: Ponerinae): new records from the Southern Hemisphere." Neotrop. Entomol. 31(1): 149-151.
15. Seifert, B. (2013). "Hypoponera ergatandria (Forel, 1893) - a cosmopolitan tramp species different from H. punctatissima (Roger, 1859) (Hymenoptera: Formicidae)." Soil Organisms 85(3): 189-201
16. Bertelsmeier et al. (2017) Recent human history governs global ant invasion dynamics. Nature Ecology & Evolution. 1:1-5.
17. Fournier et al. (2018) Predicting future invaders and future invasions. PNAS 116 (16) 7905-7910;
18. Seifert (2018). The ants of Central and North Europe. lutra Verlags und Vertriebsgesellschaft, , 408 pp. Tauer, Germany
19. Bernard (1986). Les fourmis (Hymenoptera, Formicidae) d'Europe Occidentale et Septentrionale. Faune de l'Europe et du Bassin Méditerranéen 3. 400 pp. Masson et Cie Ed.

**Table S3.** Outputs from the principal component analyses (PCA) of the seven bioclimatic variables for all ant species. The variance explained by each of the first two axes and the contributions of the original variables to these axes are given. The values correspond to the squared correlation coefficients.

| Axis | 1 | 2 |
| --- | --- | --- |
| Variance explained (%) | 61.6 | 22.7 |
| Mean annual temperature | -0.96 | 0.18 |
| Mean diurnal range | -0.53 | -0.79 |
| Temperature seasonality | 0.28 | -0.75 |
| Mean temperature of the wettest quarter of the year | -0.60 | 0.57 |
| Annual precipitation | 0.93 | 0.16 |
| Precipitation of driest month | 0.97 | 0.16 |
| Precipitation seasonality | -0.93 | 0.00 |

**Figure S1.** Map of the study zone for which species records were obtained. Map has been created by combining species records and the object wrld_simple in the *maptools* package in R (R Core Team 2016, URL <http://www.R-project.org/>).


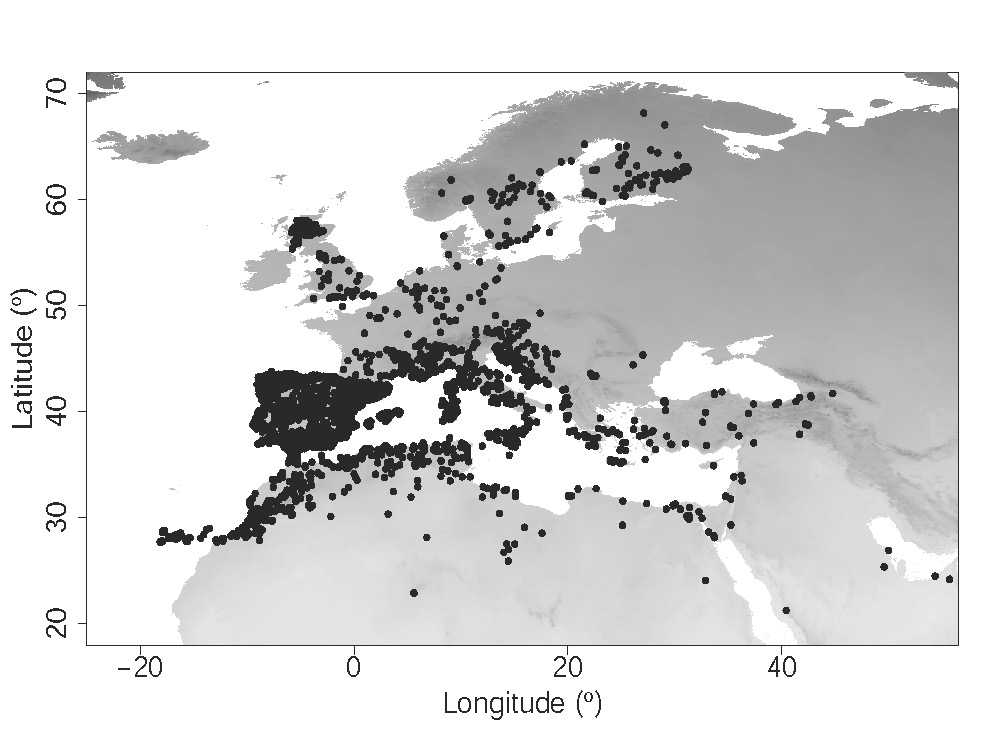


**Figure S2.** Maps of species records of the five ant species found in our study area that have been introduced out of its native range somewhere in Europe (i.e. N. Europe). We show the locations used, confirming that any of the introduced locations is comprised in our dataset. More information in Table S2. Maps have been created by combining species records and the object wrld_simple in the *maptools* package in R (R Core Team 2016, URL <http://www.R-project.org/>).


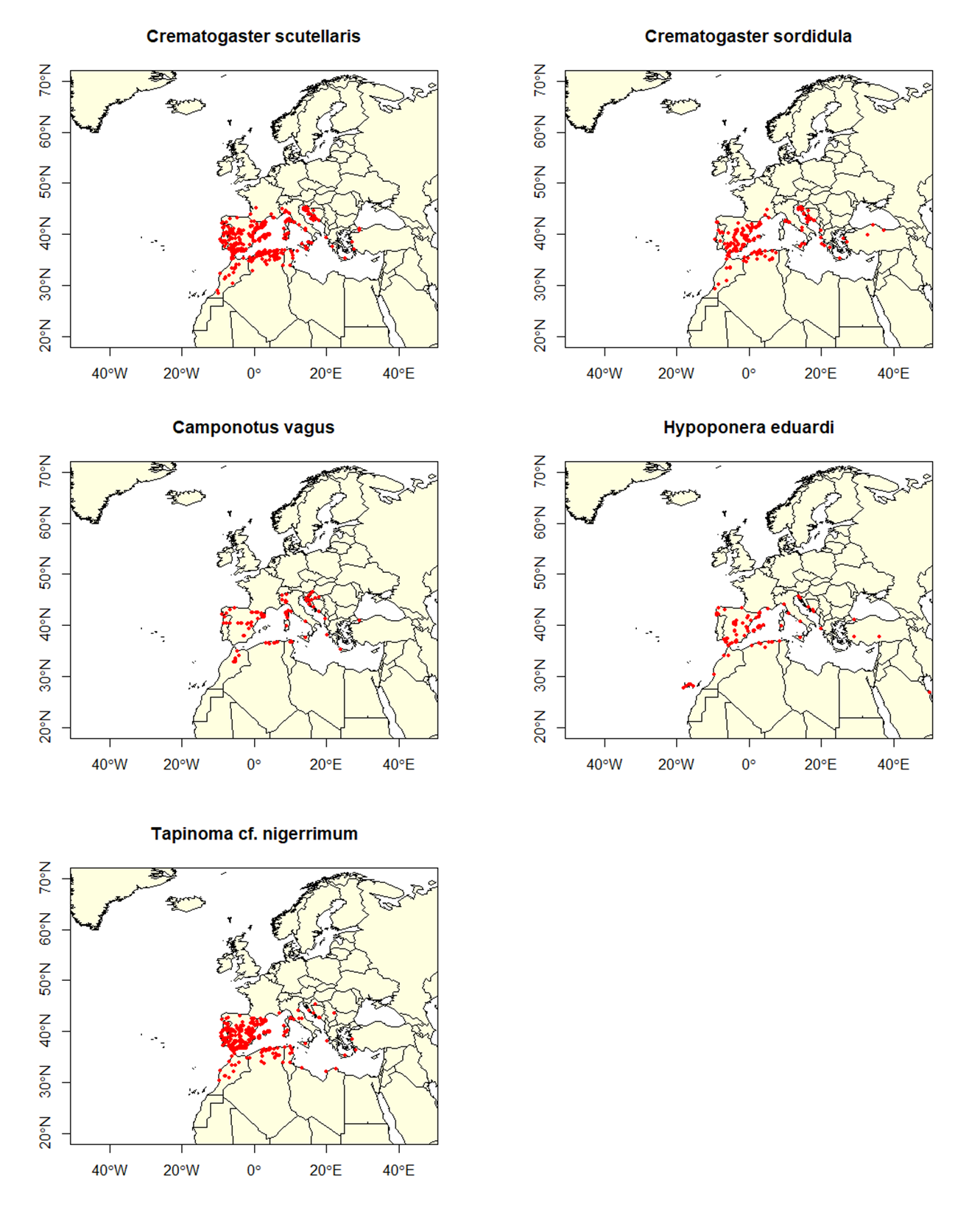


**Figure S3.** Ant phylogeny used in this study. Introduced species are highlighted with a red arrow.


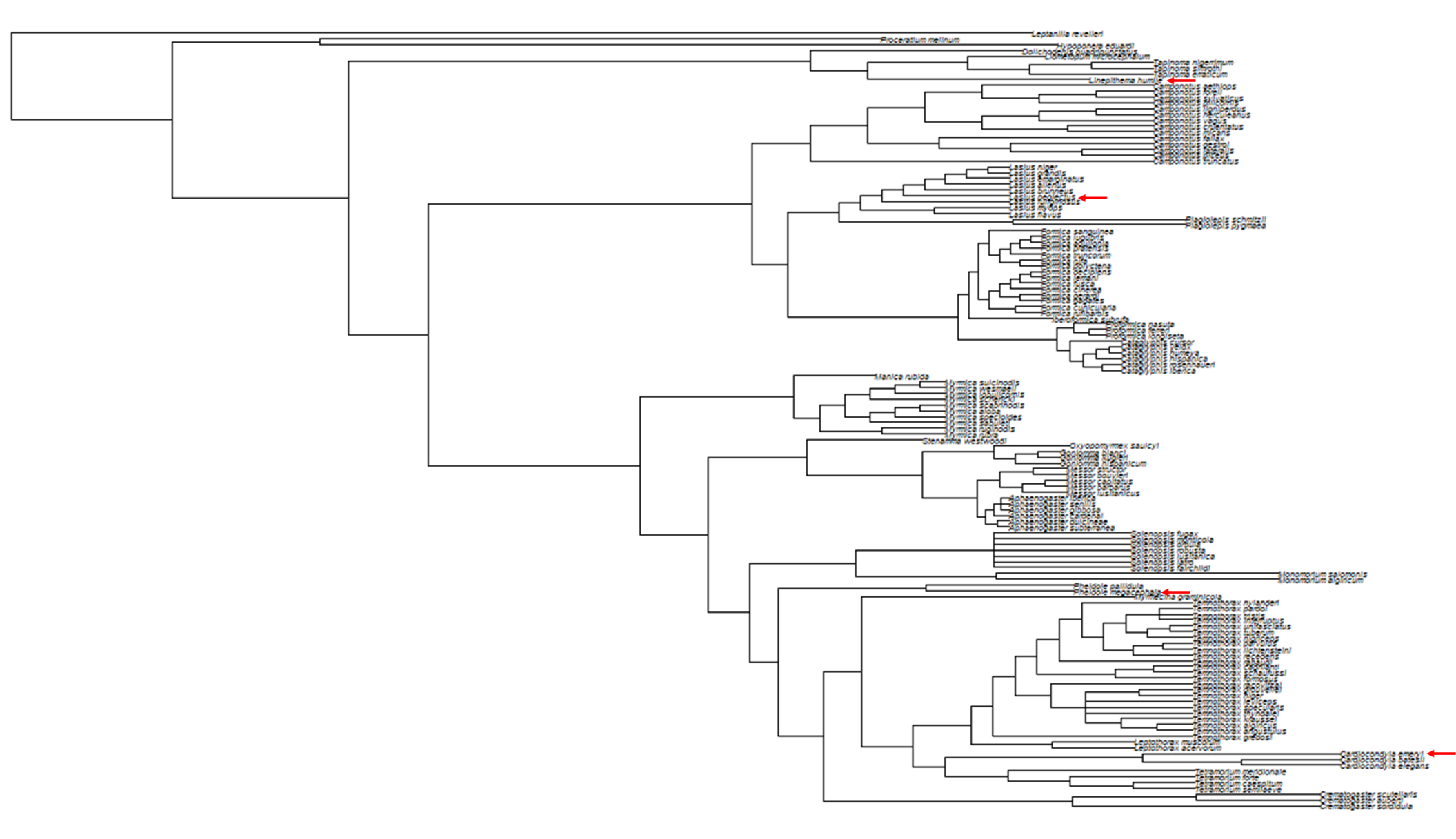


**Figure S4.** Species records for the four introduced species and their phylogenetically closest relative species. Maps have been created by combining species records and the object wrld_simple in the *maptools* package in R (R Core Team 2016, URL <http://www.R-project.org/>).

**
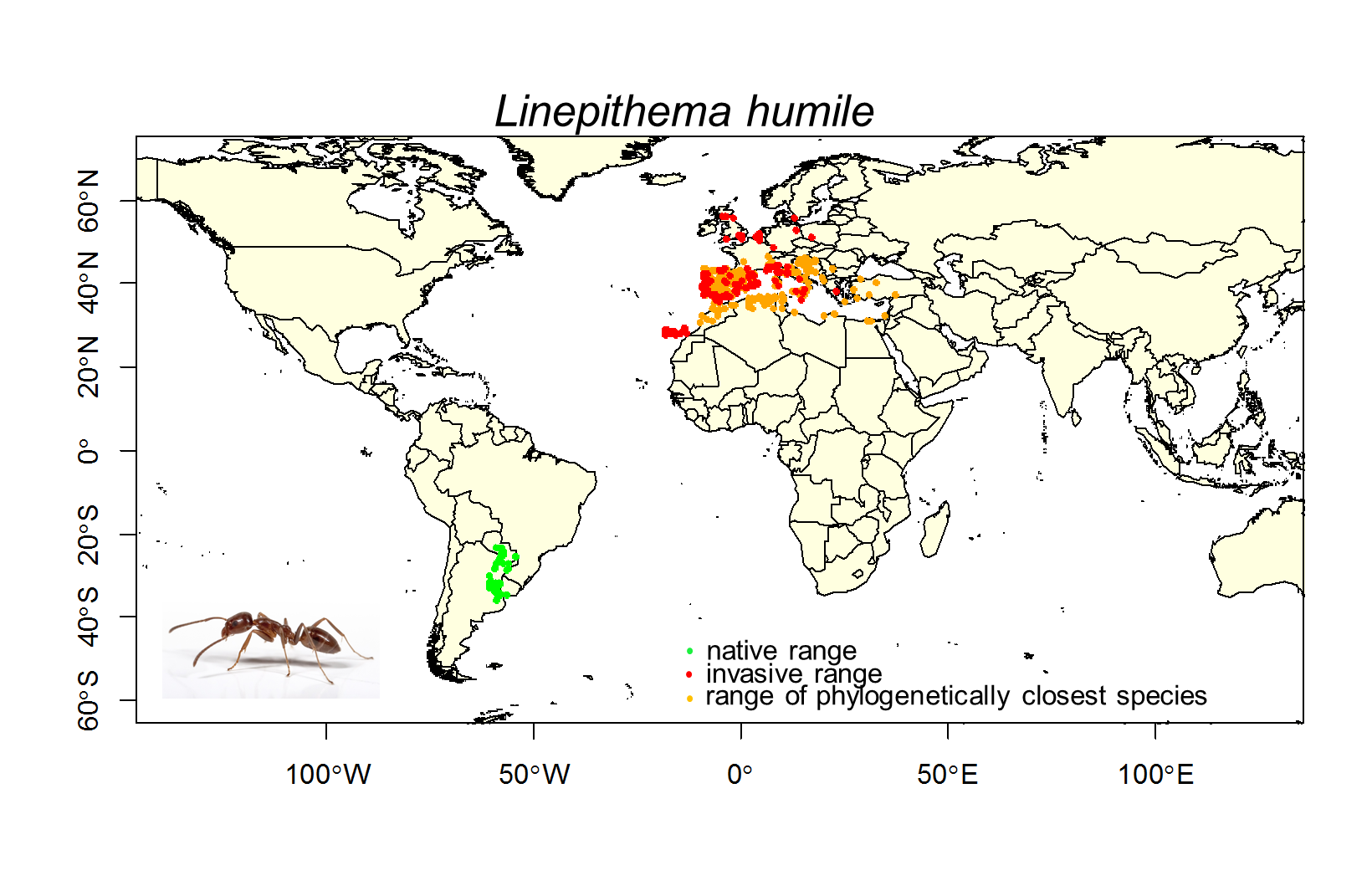
**

**
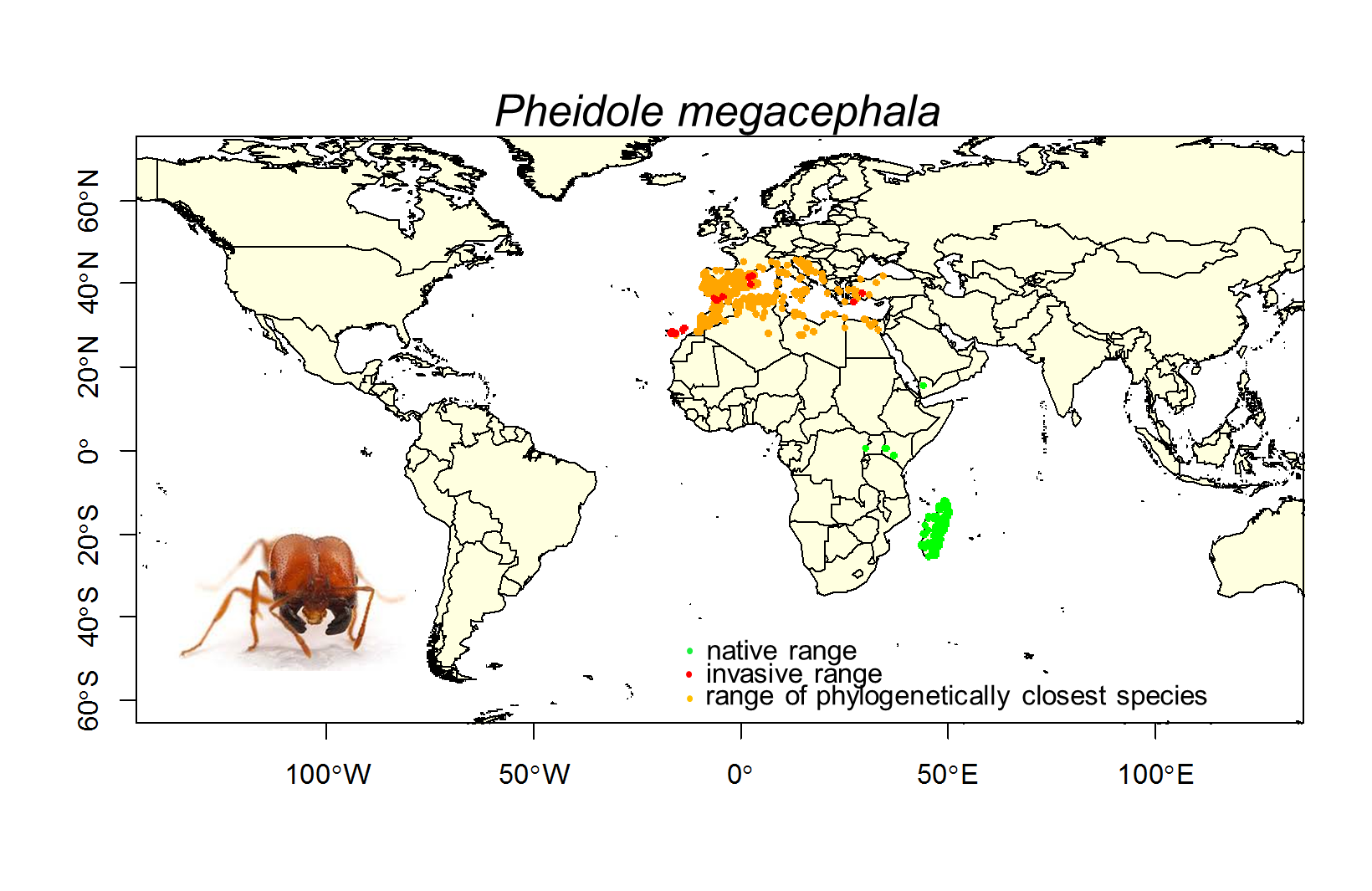
**

**
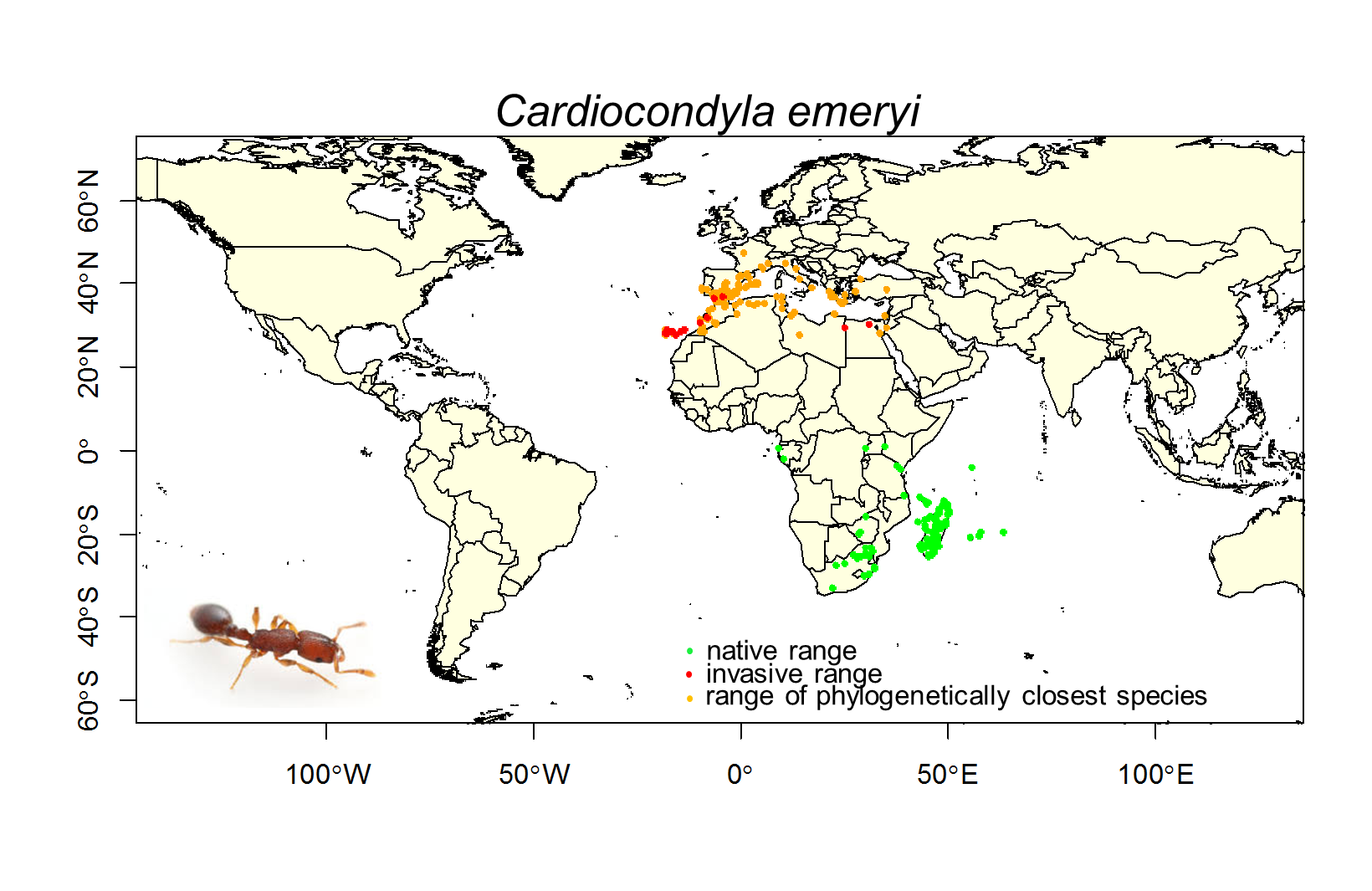
**

**
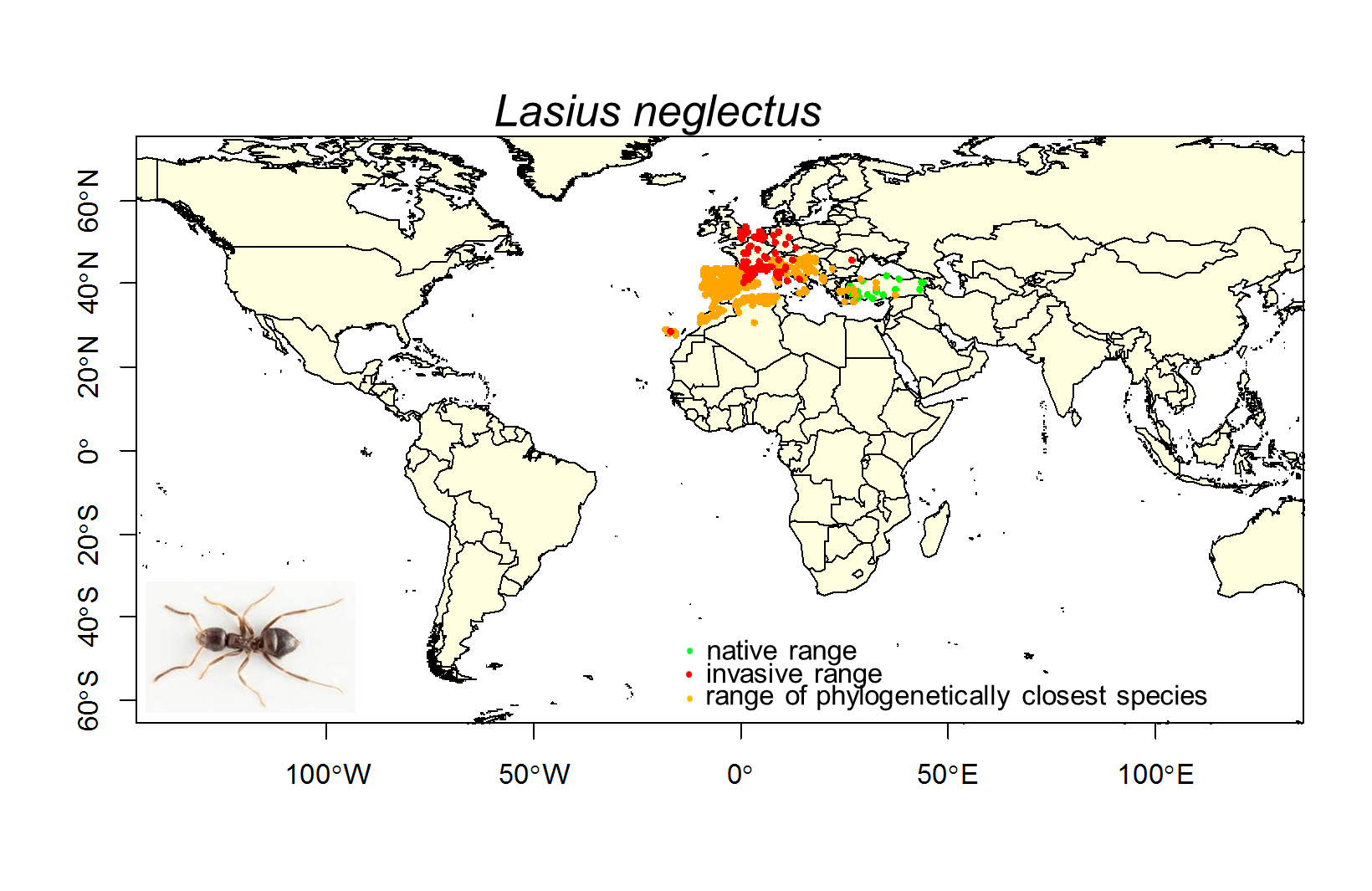
**

**Figure S5.** Plot of within-groups sum of squares against number of clusters using the k-means approach in order to choose the number of clusters for the PCA.**
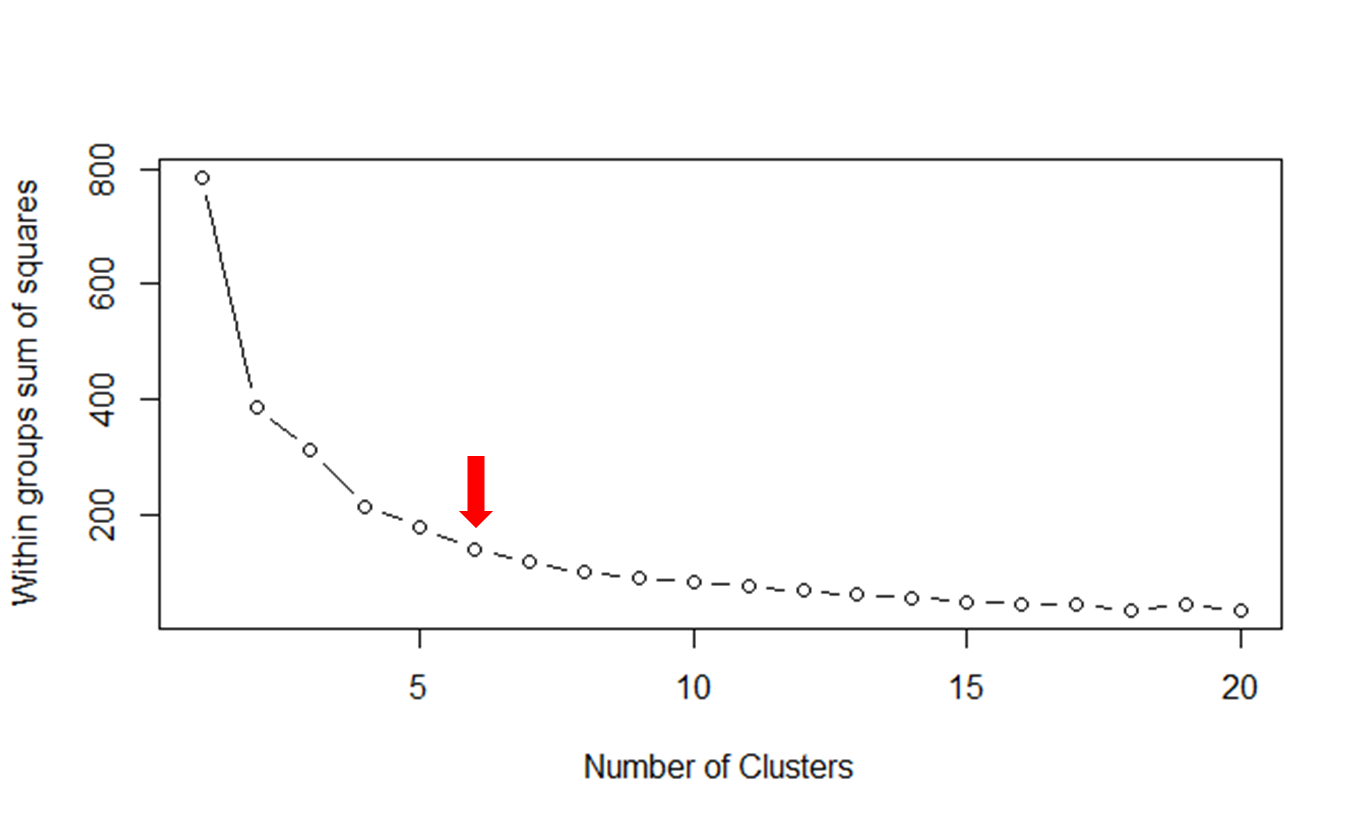
**

**Appendix S1.** List of references utilized to build the working phylogeny for the 134 European ant species examined in this study. References that used molecular data are indicated as *.

(many of them have been obtained from AntWeb. Available from http://www.antweb.org. Accessed 10 January 2014)

Agosti D (1990) Review and reclassification of *Cataglyphis* (Hymenoptera, Formicidae). Journal of Natural History 24:1457-1505

* Beibl J, Buschinger A, Foitzik S, Heinze J (2007) Phylogeny and phylogeography of the Mediterranean species of the parasitic ant genus *Chalepoxenus* and its *Temnothorax* hosts. Insectes Sociaux 54:189-199

Bernard F 1950 ("1946"). Notes sur les fourmis de France. II. Peuplement des montagnes méridionales. Annales de la Société Entomologique de France 115:1-36

Bernard F (1968) Les Fourmis (Hymenoptera Formicidae) d'Europe Occidentales et Septentrionale. Masson et Cie éditeurs, Paris. 411pp.

* Brady S.G., Schultz T.R., Fisher B.L., Ward P.S. (2006) Evaluating alternative hypotheses for the early evolution and diversification of ants. PNAS 103: 18172-18177.

* Brunner E, Kroiss J, Trindl A, Heinze J (2011) Queen pheromones in *Temnothorax* ants: control or honest signal? BMC Evolutionary Biology 11:55. http://www.biomedcentral.com/1471-2148/11/55

Buschinger A (1966) *Leptothorax* (*Mychothorax*) *muscorum* Nylander und *Leptothorax* (*M.)* *gredleri* Mayr zwei gute Arten. Insect Soc 13: 165-172.

Cagniant H, Espadaler X (1997). Les *Leptothorax*, *Epimyrma* et *Chalepoxenus* du Maroc (Hymenoptera: Formicidae). Clé et catalogue des espèces. Annales de la Société Entomologique de France (NS) 33: 259-284

Espadaler X (1996) Diagnosis preliminar de siete especies nuevas de hormigas de la Península Ibérica (Hymenoptera: Formicidae). Zapateri 6: 151-153

* Goropashnaya AV, Fedorov VB, Seifert B, Pamilo P (2012) Phylogenetic relationships of palaearctic *Formica* species (Hymenoptera, Formicidae) based on mitochondrial cytochrome b sequences. PLoS ONE 7(7): e41697. doi:10.1371/journal.pone.0041697

Janda M., Folkova D., Zrzavy J. (2004) Phylogeny of *Lasius* ants based on mitochondrial DNA and morphology, and the evolution of social parasitism in the Lasiini (Hymenoptera: Formicidae). Molecular Phylogenetics and Evolution 33: 595-614.

* Jansen G, Savolainen R, Vepsäläinen K (2009) DNA barcoding as a heuristic tool for classifying undescribed Nearctic *Myrmica* ants (Hymenoptera: Formicidae). Zoologica Scripta 38: 527-536.

* Jowers M.J., Amor F., Ortega P., Lenoir A., Boulay R.R., Cerdá X., Galarza J.A. (2014) Recent speciation and secondary contact in endemic ants. Molecular Ecology 23: 2529-2542.

* Knaden M., Tinaut A., Stökl J., Cerdá X., Wehner R. (2012) Molecular phylogeny of the desert ant genus *Cataglyphis* (Hymenoptera: Formicidae). Myrmecol. News 16: 123-132.

* Machac A, Janda M, Dunn RR, Sanders NJ (2011) Elevational gradients in phylogenetic structure of ant communities reveal the interplay of biotic and abiotic constraints on species density. Ecography 34: 364-371.

* Maruyama M, Steiner FM, Stauffer C, Akino T, Crozier RH, Schlick-Steiner BC (2008) A DNA and morphology based phylogenetic framework of the ant genus *Lasius* with hypotheses for the evolution of social parasitism and fungiculture. BMC Evolutionary Biology 8:237. doi:10.1186/1471-2148-8-237.

* Moreau CS, Bell CD, Vila R, Archibald SB, Pierce NE (2006) Phylogeny of the ants: diversification in the age of angiosperms. Science 312:101-104.

* Moreau C.S., Bell C.D. (2013) Testing the museum versus cradle tropical biological diversity hypothesis: phylogeny, diversification, and ancestral biogeographic range evolution of the ants. Evolution 67: 2240-2257.

* Muñoz-López M., Palomeque T., Carrillo J.A., Pons J., Tinaut A., Lorite P. (2012) A new taxonomic status for *Iberoformica* (Hymenoptera, Formicidae) based on the use of molecular markers. J Zool Syst Evol Res 50: 30-37.

* Oettler J, Suefuji M, Heinze J (2010) The evolution of alternative reproductive tactics in *Cardiocondyla* male ants. Evolution 64: 3310-3317

Radchenko A.G., Elmes G.W. (2004) Taxonomic notes on the *scabrinodis*-group of *Myrmica* species (Hymenoptera: Formicidae) living in eastern Europe and western Asia, with a description of a new species from Tien Shan. Proc.Russian Entomol. Soc. St. Petersburg 75: 222-233.

Rigato F (2011) Contributions to the taxonomy of West European and North African *Stenamma* of the *westwoodii* species-group. (Hymenoptera Formicidae). Memorie della Società Italiana di Scienze Naturali e del Museo Civico di Storia Naturale di Milano 37: 1-56.

Sanetra M, Güsten R, Schulz A (1999) On the taxonomy and distribution of the Italian *Tetramorium* species and their social parasites (Hymenoptera Formicidae). Memorie della Società Entomologica Italiana 77: 317-357.

* Sauer C., Stackebrandt E., Gadau J., Hölldobler B., Gross R. (2000) Systematic relationships and cospeciation of bacterial endosymbionts and their carpenter ant host species: proposal of the new taxon *Candidatus* *Blochmannia* gen. nov. Intern.J.Syst.Evol.Microb. 50: 1877-1886.

* Schlick-Steiner BC, Steiner FM, Moder K, Seifert B, Sanetra M, Dyreson E, Stauffer C, Christian E (2006) A multidisciplinary approach reveals cryptic diversity in western Palearctic *Tetramorium* ants (Hymenoptera: Formicidae). Molecular Phylogenetics and Evolution 40: 259-273.

* Schlick-Steiner B.C., Steiner F.M., Konrad H., Markó B., Csösz S., Heller G., Ferencz B., Sipos B., Christian E., Stuffer C. (2006) More than one species of *Messor* harvester ants (Hymenoptyera: Formicidae) in Central Europe. Eur. J. Entomol. 103: 469-476.

* Schmidt C.A. (2013) Molecular phylogenetics of ponerine ants (Hymenoptera: Formicidae: Ponerinae). Zootaxa 3647: 201-250.

Seifert B (1992) A taxonomic revision of the Palaearctic members of ant subgenus *Lasius* s.str. (Hymenoptera: Formicidae). Abh. Ber. Naturkundemus. Görlitz 66: 1-67.

Seifert, B. (2000) *Myrmica lonae* Finzi, 1926 – a species separate from *Myrmica sabuleti* Meinert, 1861 (Hymenoptera: Formicidae). Abh. Ber. Naturkundemus. Görlitz 72, 195–205.

Seifert, B., Schultz R (2009) A taxonomic revision of the *Formica rufibarbis* Fabricius, 1793 group (Hymenoptera: Formicidae). Myrmecological News 12: 255-272.

Tinaut A (1991) [1990]. Situación taxonómica del género *Cataglyphis* Förster, 1850 en la Península Ibérica. III. El grupo de *C. velox* Santschi, 1929 y descripción de *Cataglyphis humeya* sp. n. (Hymenoptera, Formicidae). EOS 66: 215-227

Tinaut A (1993) *Cataglyphis floricola* nov. sp. new species for the genus *Cataglyphi*s Förster, 1850 (Hymenoptera, Formicidae) in the Iberian Peninsula. Mitt Schweiz Entomol Ges 66: 123-134

Tinaut A, Ruano F, Hidalgo J, Ballesta M (1994) Mirmecocenosis del sistema de dunas del Paraje Natural Punta Entinas-El Sabinar (Almería) (Hymenoptera Formicidae) Aspectos taxonómicos functionales y biogeográficos. Graellsia 50: 71-84

Ward PS (2007) Phylogeny, classification, and species-level taxonomy of ants (Hymenoptera: Formicidae). Zootaxa 1668: 549-563

**Appendix S2.** Selection of climatic predictor variables

Climate datasets were compiled from monthly data available from the WorldClim 2.0 database (Hijmans et al. 2005) for each point where a species was recorded. We discarded the BIO7 variable (temperature annual range=BIO5-BIO6) from the original set of 19 WorldClim variables because BIO7 is simply a linear combination of BIO5 and BIO6. Next, we used functions from the “raster” (Hijmans 2015) and “dismo” (Hijmans et al. 2011) packages of the R software (R Core Team, 2015) to retrieve and build the set of climate variables, respectively. To accomplish an acceptable gain in computational efficiency and memory we sought to achieve a sensible reduction in predictor variable dimensionality, while, at the same time, preserving a good approximation to the original set of variables. We first discarded substituting the climatic variables by their decomposition via principal component analysis (PCA) because we would not be able to easily identify the resulting PCA components with meaningful climatic variables. Therefore, we chose the approach by Cadima and Jolliffe (2001) to approximate the whole set of 19 climatic variables with a smaller subset. To that goal we used the function “eleaps” from the “subselect” (Orestes Cerdeira et al. 2015) package in R. In the options to the “eleaps” function we selected the criterion “RM” for variable selection ($r_{m}$ in Eq. 2.8 of Cadima and Jolliffe 2001), which maximized the $r_{m}$ index in Eq. 2.8 of Cadima and Jolliffe (2001). This index can be interpreted as the square root of the percentage of the total variance accounted for by the variable subset.

Before calculating and selecting the subset of climatic variables we forced the algorithm to pre-select two variables, BIO1 (annual mean temperature) and BIO12 (annual precipitation), such they were automatically included within the final subset. We considered these two variables to be necessary drivers that should not be discarded by the algorithm in the “eleaps” function. We then set $r_{m}=0.99$, which allowed us to reduce our initial set of climatic variables from 18 down to 7. The resulting variables were: BIO1 (annual mean temperature), BIO2 (mean diurnal range), BIO4 (temperature seasonality), BIO8 (mean temperature of wettest quarter), BIO12 (annual precipitation), BIO14 (precipitation of driest month) and BIO15 (precipitation seasonality). They thus included 4 temperature-based and 3 precipitation-based variables. For a full account of definitions and methodologies of these climatic variables see http://www.worldclim.org and references therein.

References:

Cadima, J., Jolliffe, I. (2001). Variable selection and the interpretation of principal subspaces. Journal of Agricultural, Biological and Environmental Statistics, 6(1), 62-79.

Hijmans, R.J., Cameron, S.E., Parra, J.L., Jones, P.G. & Jarvis, A. (2005) Very high resolution interpolated climate surfaces for global land areas. International Journal of Climatology, 25, 1965-1978.

Hijmans, R.J. (2015). raster: Geographic Data Analysis and Modeling. R package version 2.5-2. https://cran.r-project.org/package=raster.

Hijmans, R.J., Phillips, S., Leathwick, J., Elith, J. (2011). dismo: Species Distribution Modeling. R package version 1.0-15. <https://cran.r-projecto.org/package=dismo>.

Orestes Cerdeira, J., Duarte Silva, P., Cadima, J., Minhoto, M. (2015). subselect: Selecting Variable Subsets. R package versión 0.12-5. <https://cran.r-project.org/package=subselect>

R Core Team (2015). R: A language and environment for statistical computing. R Foundation for Statistical Computing, Vienna, Austria. <https://www.r-project.org/>.
